# Molecular biomarkers involved in the progression of gallbladder inflammatory lesions to invasive cancer: A proteomic approach

**DOI:** 10.17305/bb.2024.10704

**Published:** 2024-09-08

**Authors:** Neetu Rawal, Gururao Hariprasad, Sabyasachi Bandyopadhyay, Nihar Ranjan Dash, Sunil Kumar, Prasenjit Das, Sharmistha Dey, Maroof Ahmad Khan, Amar Ranjan, Anita Chopra, Sundeep Saluja, Showket Hussain, GK Rath, Tanvir Kaur, Pranay Tanwar

**Affiliations:** 1 Laboratory Oncology Unit, Dr. B.R.A. Institute Rotary Cancer Hospital, All India Institute of Medical Sciences, New Delhi, India; 2 Department of Biophysics, All India Institute of Medical Sciences, New Delhi, India; 3 Proteomics Laboratory, Centralized Core Research Facility, All India Institute of Medical Sciences, New Delhi, India; 4 Department of Gastrointestinal Surgery, All India Institute of Medical Sciences, New Delhi, India; 5 Department of Surgical Oncology, All India Institute of Medical Sciences, New Delhi, India; 6 Department of Pathology, All India Institute of Medical Sciences, New Delhi, India; 7 Department of Biostatistics, All India Institute of Medical Sciences, New Delhi, India; 8 Department of Gastrointestinal Surgery, Govind Ballabh Pant Hospital, New Delhi, India; 9 Division of Molecular Oncology, National Institute of Cancer Prevention and Research, Indian Council of Medical Research, New Delhi, India; 10 Dr. B.R.A. Institute Rotary Cancer Hospital, All India Institute of Medical Sciences, New Delhi, India; 11 Division of Non-Communicable Diseases, Indian Council of Medical Research, New Delhi, India

**Keywords:** Liquid chromatography-assisted tandem mass spectrometry, biomarker, differentially expressed proteins, gallbladder cancer, ELISA, real time-polymerase chain reaction, inflammatory lesion

## Abstract

The progression of gallbladder inflammatory lesions to invasive cancer remains poorly understood, necessitating research on biomarkers involved in this transition. This study aims to identify and validate proteins associated with this progression, offering insights into potential diagnostic biomarkers for gallbladder cancer (GBC). Label-free liquid chromatography-assisted tandem mass spectrometry (LC-MS/MS) proteomics was performed on samples from ten cases each of GBC and inflammatory lesions, with technical duplicates. Validation was conducted through the enzyme-linked immunosorbent assay (ELISA) using 80 samples (40 GBC and 40 inflammatory lesions). Bioinformatics tools analyzed protein–protein interaction (PPI) networks and pathways. Statistical correlations with clinicopathological variables were assessed. Prognostic evaluation utilized Kaplan–Meier survival analysis and Cox regression analyses. mRNA expressions were studied using real-time-polymerase chain reaction (RT-PCR). Out of 5714 proteins analyzed, 621 were differentially expressed. Three upregulated (the S100 calcium-binding protein P [S100P], polymeric immunoglobulin receptor [PIGR], and complement C1q-binding protein [C1QBP]) and two downregulated (transgelin [TAGLN] and calponin 1 [CNN1]) proteins showed significant expression. Pathway analysis implicated involvement of proteoglycans in cancer and glycosaminoglycan metabolism. Significant correlations were observed between protein concentrations and clinicopathological variables. Prognostic factors, such as tumor size, lymph node metastasis, and preoperative bilirubin levels were associated with overall survival (OS). Protein-based assays demonstrated higher resolution compared to mRNA analysis, suggesting their utility in GBC risk stratification. S100P, PIGR, C1QBP, TAGLN, and CNN1 emerge as potential protein-based biomarkers involved in the progression from gallbladder inflammatory lesions to invasive cancer. These findings hold promise for improved diagnostic and prognostic strategies in GBC management.

## Introduction

Gallbladder cancer (GBC) is a relatively rare but aggressive malignancy that accounts for approximately 165,000 (1.7%) cancer deaths annually worldwide [1, 2]. The greatest incidence rates of GBC are found in Chile (27 per 100,000 people), followed by northern India at 21.5 per 100,000 people. In India, GBC occurrence is significantly higher in the northern regions compared to the southern states, with rates of 8.9 per 100,000 in Delhi compared to 0.8 per 100,000 in Chennai [3, 4]. The gallbladder is a small cystic organ located under the inferior surface of the liver. Its primary function is to store and concentrate bile produced by the liver and transport it to the small intestine via the cystic duct [1]. The gallbladder consists of several layers: (a) the mucosa (innermost layer of epithelial cells), (b) the muscular layer (smooth muscle cells), (c) the perimuscular layer (connective tissue), and (d) the serosa (outer layer). Under the influence of gallstones and other carcinogenic insults, the normal gallbladder epithelial layer undergoes metaplastic changes, which can lead to dysplasia, carcinoma in situ (CIS), and eventually invasive carcinoma. The multistage pathogenesis of gallbladder carcinoma arises due to prolonged exposure to gallstones (cholelithiasis), creating an inflammatory environment known as chronic cholecystitis, which increases the risk of GBC [5, 6]. The most common subtype of GBC is adenocarcinoma, accounting for 80% to 97% of cases, originating from secretory cells. Other subtypes include papillary, mucinous, squamous, and adenosquamous carcinoma. Of these, the papillary subtype is the rarest, but it has a better prognosis compared to other subtypes [7].

Several risk factors for GBC include ethnicity, age, sex, chronic inflammation, gallstones, infections, exposure to heavy metals and environmental toxins, obesity, gallbladder polyps, genetic predispositions, and abnormalities in the pancreaticobiliary ductal junction [8]. To date, no diagnostic marker is available for the early detection of GBC. While combinations of markers, such as CEA, CA125, CA242, and CA19-9, are used for diagnosing liver, gastric, colorectal, and pancreatic cancers, these markers have shown less specificity and sensitivity when tested in GBC, yielding inconsistent results. Therefore, they cannot be used as standalone diagnostic markers for GBC [9, 10]. Identifying GBC in its early stages is challenging, despite advancements in ultrasound and computed tomography (CT) scanning. Only half of gallbladder cancers are diagnosed prior to surgical intervention. Diagnosis still relies on clinical assessment, followed by imaging-guided fine needle aspiration cytology (FNAC) [11]. Radical surgical extirpation remains the only effective treatment, as most cases present at an advanced stage due to missed early detection. This is partly because the gallbladder lacks a submucosal layer to limit the spread of cancer [12]. The progression from inflammatory lesions to invasive cancer occurs through metaplasia–dysplasia–carcinoma due to recurrent epithelial damage.

Thus, there is an urgent need for research on biomarkers involved in the progression of gallbladder inflammatory lesions to invasive cancer. This study used comparative protein profiling between two clinical phenotypes of the gallbladder (cancer and inflammatory lesion) to identify differentially expressed proteins (DEPs) that may be associated with the development of GBC in the context of pre-existing inflammation.

## Materials and methods

### Sample collection

A questionnaire was used to gather clinical information from the patients. Tissues were collected from the resected gallbladder of patients during surgery, placed in a 1X PBS vial, and immediately stored at −80 ^∘^C. The collected tissue was histopathologically examined for phenotype confirmation before further analysis. The percentage of tumor cells in each tissue sample was carefully assessed and recorded. Samples containing more than 70% tumor cells in GBC cases were selected for further analysis.

### Inclusion and exclusion criteria of patients

The staging of GBC was determined according to the American Joint Committee on Cancer (AJCC) cancer staging criteria. Patients were included in the study based on the following inclusion criteria: (a) Patients diagnosed with any type of GBC and (b) Patients diagnosed with inflammatory lesions of the gallbladder. The exclusion criteria were: (a) Patients who had received chemotherapy or radiation therapy.

### Protein isolation and quantification

**Figure 1.**
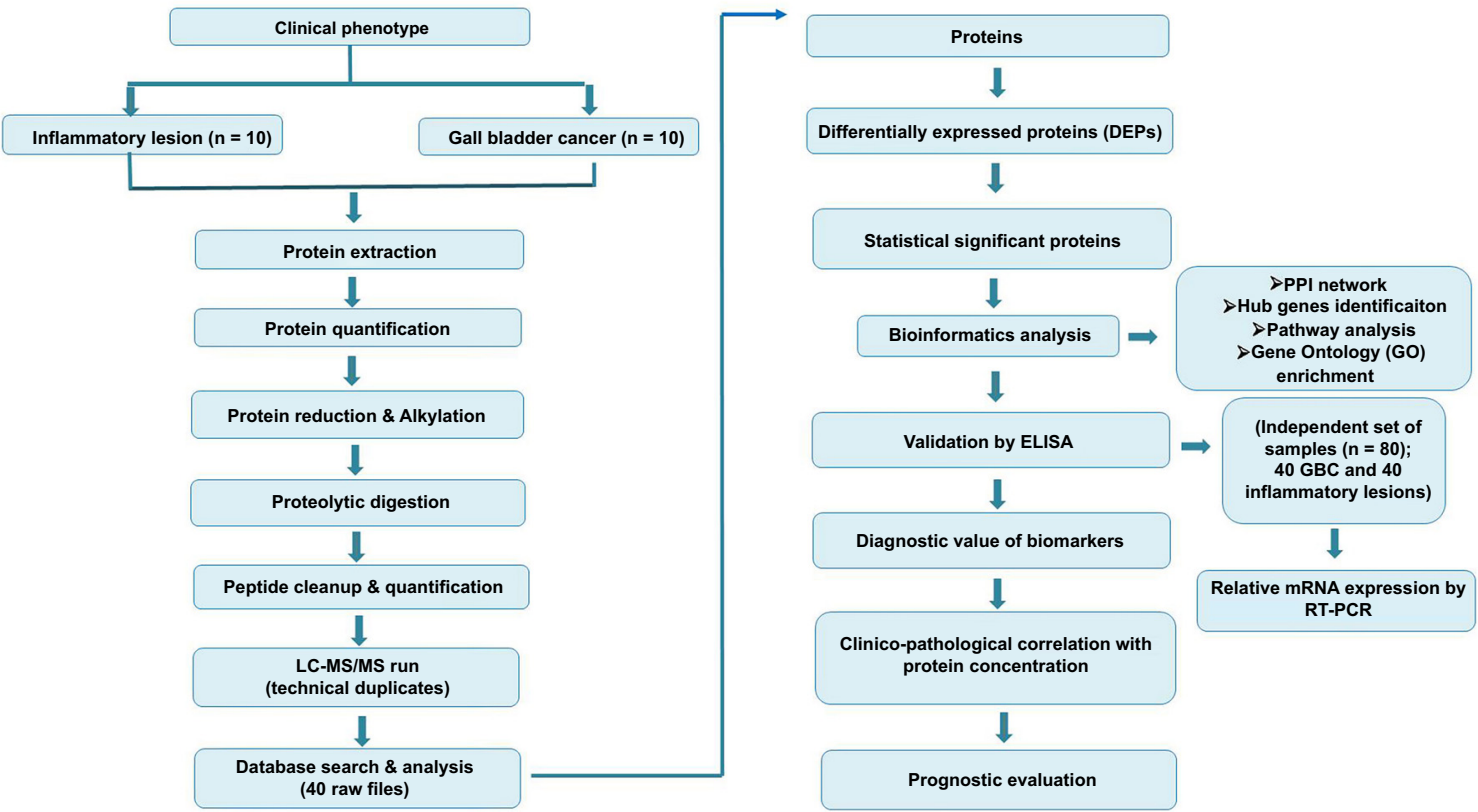
**Study workflow.** LC-MS/MS: Label-free liquid chromatography-assisted tandem mass spectrometry.

Homogeneous samples from two clinical phenotypes of the gallbladder, including stage 2 adenocarcinoma (*n* ═ 10) and chronic cholecystitis lesions, were used for the discovery phase of proteomic experiments via Liquid Chromatography–Tandem Mass Spectrometry (LC-MS/MS) (Label-free). The study design is illustrated in (Figure 1). Protein was isolated using EasyPrep lysis buffer (Thermo Scientific, Catalog no. A45735). Approximately 5 mg of gallbladder tissue was homogenized with 100 µL of prewarmed lysis buffer, followed by the addition of 1 µL of universal nuclease enzyme (Thermo Scientific, Catalog no. 88700). The homogenized tissue was centrifuged at 16,000 *g* for 10 min, and the supernatant was collected in a vial. Protein concentration was determined using a UV–Vis spectrophotometer (Thermo Scientific, Nanodrop™) at an A280 nm wavelength with baseline correction at 340 nm.

### Protein digestion and peptide cleanup

A total of 25 µg of isolated protein was used for the protein digestion protocol (Thermo, Mini MS Sample kit, Catalog no. A40006). 50 µL of reduction solution and 50 µL of alkylation solution were added to the protein sample, followed by incubation at 95 ^∘^C for 10 min. For protein digestion, 50 µL of Trypsin/Lys-C protease mix was added to the reduced and alkylated sample and incubated overnight at 37 ^∘^C. The digestion reaction was stopped with a stop solution. The peptide cleanup protocol was then followed as per the kit manual. Briefly, the digested protein sample was transferred into a peptide desalting column and centrifuged. The flow-through was discarded, and the column was washed twice with a wash solution. Peptides were eluted and dried by lyophilization. The sample was resuspended in 0.1% formic acid for LC-MS/MS analysis.

### Liquid chromatography-assisted tandem mass spectrometry (LC-MS/MS)

In the discovery phase, a total of 20 homogeneous gallbladder tissue samples were used in each phenotype group. Ten samples of GBC (stage 2, adenocarcinoma) and ten samples from inflammatory lesions (chronic cholecystitis) were subjected to a label-free LC-MS/MS experiment in technical duplicates. LC-MS/MS analysis was performed using an Orbitrap Fusion Tribrid mass spectrometer (Thermo Scientific, USA) connected to an ultra-pressure nano-flow liquid chromatography system. 1 µg of peptide mixture was injected onto a reverse-phase (RP) trap column, Acclaim PepMap 100 C18 (75 µm × 2 cm, 3 µm, 100 Å; Thermo Fisher Scientific), at a flow rate of 300 nl/min. LC-MS grade water containing 0.1% FA was used as the loading buffer, and 0.1% FA and 80% ACN in LC-MS grade water were used as the elution buffer. Peptides were washed with the loading buffer for 45 min to eliminate excess salt. Subsequently, the retained peptides were separated on an RP analytical column, Acclaim PepMap 100 C18 (75 µm × 15 cm, 2 µm, 100 Å; Thermo Fisher Scientific), prior to connection to the mass spectrometer. The elution gradient started at 5% elution buffer, gradually increasing at a linear rate to 8% over 5 min, then to 60% over 110 min, and finally to 95% over 2 min. The gradient was maintained at 95% elution buffer for 5 min before re-equilibration with 5% elution buffer for 20 min. Throughout the LC-MS/MS analysis, the loading buffer was used as a blank, while a tryptic-digested HeLa standard (200 ng) served as the QC standard. A total of 1-µg pooled peptide sample acted as the control for the LC-MS/MS run. The mass spectrometer operated in data-dependent acquisition (DDA) mode. Full MS spectra were collected in positive ionization mode, with an ion-spray voltage of 2100 V and an *m*/*z* range of 350–2000 Da, using a 50-ms injection time. At the MS1 level, precursor/peptide isolation used a quadrupole ion filter and an Orbitrap mass analyzer, set at a resolution of 60,000 (at 200 *m*/*z*) and an AGC target of 1e6. The top 20 precursors were selected for fragmentation (MS2 or MS/MS) via a linear ion trap, with a resolution setting of approximately 30,000 (at 200 *m*/*z*) and an AGC target of 1e5. DDA incorporated advanced “rolling collision energy” for subsequent MS/MS scans, with the normalized high-energy collision-induced dissociation (HCD) fragmentation energy fixed at 30%.

### Database search and analysis

The precision and accuracy of the raw data generated through the LC-MS/MS instrument were verified using X Caliber software. All 40 files obtained were normalized and analyzed using Proteome Discoverer 2.4 SP1 (Thermo Scientific) [13]. This software enabled the collective analysis of all raw files, facilitating the identification of DEPs with more than 30% coverage across all 40 samples. To ensure accuracy and identify efficient biomarkers, DEPs consistently present across all samples were filtered and selected. The data are available through the ProteomeXchange Consortium via the MassIVE data repository with the identifier PXD040704. Both the MS and MS/MS spectra were searched against the human UniProt database, appended with a list of common contaminants provided by Thermo Scientific, using the Sequest HT algorithm.

For each spectrum file, the spectrum RC node calculated a constant mass shift (ppm), and the spectrum selector node selected a subset of spectra for searching in Sequest HT. The Sequest HT parameters were specified as: trypsin enzyme, two allowed missed cleavages, minimum peptide length of 6, maximum peptide length of 144, precursor mass tolerance of 10 ppm, and fragment mass tolerance of 0.6 Da. The static modification was set to carbamidomethylation (+ 57.021 Da) of cysteine. The dynamic modification applied was methionine oxidation (+ 15.995 Da) on the peptide terminus. Additionally, N-terminal modifications were implemented: Acetyl (+ 42.011 Da), Met-loss (− 131.040 Da), and Met-loss + Acetyl (−89.030 Da).

In the Percolator node, the false discovery rate (FDR) was determined using *q*-values from the Decoy database search. Peptide spectral match filtering was conducted with a stringent FDR threshold of 0.01 and a lenient threshold of 0.05, as determined by the Percolator. Peaks and features were filtered using a Minora feature detector node, with parameters set at a minimum trace length of 5 and a maximum ΔRT of isotope pattern multiplets of 0.2. Contaminant and decoy proteins were excluded from all datasets before downstream analysis, and MSF files were processed through the consensus workflow.

In the consensus workflow, peptide spectrum matches were grouped with a site probability threshold of 75, and the peptide validator node was strictly set at a target FDR of 0.01 and relaxed at 0.05 for PSMs and peptide identification. The peptide and protein filter node was set to a minimum peptide length of 6, high peptide confidence, and a minimum of 1 peptide sequence. Only rank 1 peptides were counted as false, and only top-scored proteins were considered for the protein filter according to score thresholds. Proteins were scored and grouped, and peptides in the protein were annotated based on the position of identified peptides in the proteins found in the sample. Falsely identified proteins were filtered out, determined by the protein FDR validator node, set strictly at a target FDR of 0.01 and relaxed at 0.05.

The remaining proteins were annotated with respect to their biological processes, cellular components, and molecular functions. The final proteins were marked as master proteins along with their modification sites and peptide isoform groups. The feature mapper node, which performed retention-time alignment and feature linking across datasets, was set with the following parameters: (a) mass tolerance: 10 ppm, (b) maximum RT shift: 10 min, and (c) minimum S/N threshold: 5. The precursor ion quantifier node controlled peptide quantification based on the following parameters: (a) consider protein groups for peptide uniqueness (true), (b) precursor abundance based on intensity, (c) normalization mode of total peptide amount, (d) pairwise ratio-based protein ratio calculation, and (e) maximum fold change of 100. The log_2_ fold change of ≥1 and ≤ 1 at *P* < 0.05 was set for identifying DEPs. A volcano plot with a *P* value <0.05 was graphically generated to represent upregulated and downregulated proteins.

### Enzyme-linked immunosorbent assay (ELISA)

Protein lysates from an independent set of gallbladder tissues (*n* ═ 80; 40 cancerous and 40 inflammatory lesions) were prepared using EasyPrep lysis buffer (Thermo Scientific). All five DEPs (S100P, C1QBP, PIGR, TAGLN, and CNN1) with a log_2_ fold change of ≥2 were assayed according to the manufacturer’s protocol using commercially available ELISA kits (ELK, Catalog nos. 1290, 1865, 1752, 3895, 3478). In summary, 96-well plates were incubated at 37 ^∘^C for 80 min with standards, samples, and blank duplicates. Following incubation, the liquid was removed from the microwells, which were then washed three times with 200 µL of wash buffer. Next, 100 µL of diluted Biotin-Conjugate antibodies were added to all wells and incubated at 37 ^∘^C for 50 min. After another wash step, 100 µL of diluted Streptavidin-HRP was added and incubated for 50 min at 37 ^∘^C. Color development was initiated by adding 100 µL of TMB substrate solution to all wells and incubating at 37 ^∘^C for 20 min. Finally, 50 µL of stop reagent was added to all wells, and absorbance readings of both standards and samples were measured at 450 nm, with readings tabulated according to their dilutions.

### Pathway and protein–protein interaction (PPI) network analysis

The PPI network of the identified DEPs was generated using the STRING database (https://string-db.org/) [14], with the parameters set to a 5% FDR stringency and species specified as Homo sapiens. The significance of hub proteins in the interactome was calculated based on 12 topological methods (Closeness, Degree, Maximum neighborhood component (MNC), Maximum clique centrality (MCC), Edge percolated component (EPC), Bottleneck, EcCentricity, Density of maximum neighborhood component (DMNC), Betweeness, Radiality, Stress, and clustering coefficient) using cytoHubba, a Cytoscape plugin (https://cytoscape.org/) [15]. Pathway and Gene Ontology (GO) enrichment analyses of DEPs were conducted using DAVID (https://david.ncifcrf.gov/tools.jsp) [16], which includes three databases: KEGG, Reactome, and WikiPathways.

### RNA isolation and cDNA synthesis

Total RNA was isolated from 80 gallbladder tissues, consisting of 40 cancer cases and 40 inflammatory controls, using the RNAlater method with the Qiagen RNeasy Mini Kit, following the manufacturer’s instructions. To ensure the RNA’s suitability for downstream applications, purity and concentration were assessed using a Nanodrop spectrophotometer and a Qubit fluorometer, respectively. Additionally, the integrity of the RNA samples was confirmed using a 1.5% agarose gel and the Bioanalyzer 2100 (Agilent Technologies Inc., USA). Only samples with an RNA Integrity Number (RIN) greater than 7.0 were used for cDNA synthesis, followed by real time-polymerase chain reaction (RT-PCR). Reverse transcription was performed using the Improm II RT system from Promega to generate cDNA. Quantitative PCR was conducted using SYBR Green dye and Oligo(dT) primer on the Agilent Mx3000P qPCR Platform (Agilent, Santa Clara, CA, USA). The specific primer sequences used were as follows: S100P are Fwd: AGGTGCTGATGGAGAAGGAG, Rev: ACTCACTGAAGTCCACCTGG, C1QBP-Fwd: GGAGCTGGAACTGAATGGGA, Rev: GTTGGTGGGATGCTGTTGTT, PIGR-Fwd: Fwd: GAAAGGGCTCGGGACGATGG, Rev: TCTTCGTGGAGATGGCTGGGA, TAGLN- Fwd: GAGAGATGAGGATGGAGGCC, Rev: AGGATTGCTGCCAGAGAAGT, CNN1-Fwd: AGGTTAAGAACAAGCTGGCCC, Rev: CCGTCCATGAAGTTGTTGCC and *GAPDH* was utilized as the housekeeping gene, possessing the following sequence. of Fwd: TCGTGGAAGGACTCATGACC, Rev: ATGATGTTCTGGAGAGCCCC. Relative mRNA expression for all five genes was calculated using the 2^--ΔΔCt^ method.

### Ethical statement

The institute ethics committee at All India Institute of Medical Sciences (AIIMS), New Delhi, granted approval for this study (IECPG-608/25.11.2020, RT-25/23.12.2020). All protocols adhered to the ethical standards outlined in the Declaration of Helsinki. Patients were thoroughly informed about the clinical study before screening at the GI Surgery Department, AIIMS, New Delhi. Voluntary written informed consent was obtained from each patient before recruitment.

### Statistical analysis

Continuous variables were described using x±s and the median (range). Student’s *t*-test was applied to determine the concentrations of S100P, PIGR, C1QBP, TAGLN, and CNN1 in both cancer cases and inflammatory lesions. ROC curve analysis was used to determine the optimal cut-off value for the concentrations of these proteins, using the Youden index method [17]. The chi-square test was used to assess the association between protein expression and clinicopathological characteristics at the optimal cut-off. Student’s *t*-test was also used to assess S100P, PIGR, C1QBP, TAGLN, and CNN1 mRNA expression in cancer cases and inflammatory lesions. The Kaplan–Meier method with a log-rank test was employed to predict the overall survival (OS) of GBC cases. Prognostic factors associated with survival were evaluated through univariate and multivariate Cox proportional hazard regression analysis. All statistical analyses were performed using GraphPad Prism 8.0 and STATA version 11. *P* < 0.05 was considered statistically significant.

## Results

### Clinical profile of discovery phase

In the discovery phase, a total of 20 homogeneous gallbladder tissue samples were used for each phenotype group. Ten samples of GBC (stage 2, adenocarcinoma) and ten samples from inflammatory lesions (chronic cholecystitis) were taken for the label-free LC-MS/MS experiment. Out of the ten cancer cases, eight were female and two were male, with a median age of 56 years. None of the cancer cases had a history of gallstones. In the inflammatory lesion group, six were female and four were male, with a median age of 51 years. Three patients had a history of gallstones.

### DEP identified through LC-MS/MS

**Figure 2.**
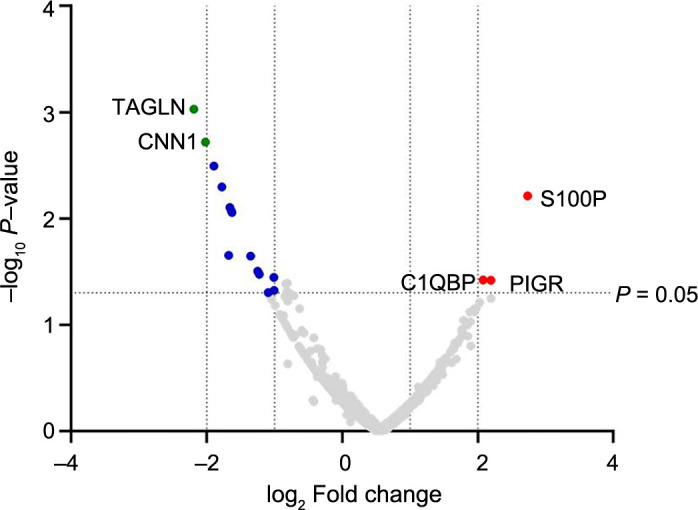
**Volcano plot of filtered proteins at Log_2_ Fold change 2.** Green colored dots represent significantly down-regulated proteins at Log_2_ fold change 2 and *P* ═ 0.05. Red-colored dots represent significantly upregulated proteins at Log_2_ fold change 2 and *P* ═ 0.05. Blue colored dots represent significantly down regulated proteins at Log_2_ fold change <2 and *P* ═ 0.05. Gray colored dots represent non-significant proteins. S100P: S100 calcium-binding protein P; PIGR: Polymeric immunoglobulin receptor; C1QBP: Complement C1q-binding protein; TAGLN: Transgelin; CNN1: Calponin 1.

**Figure S1.**
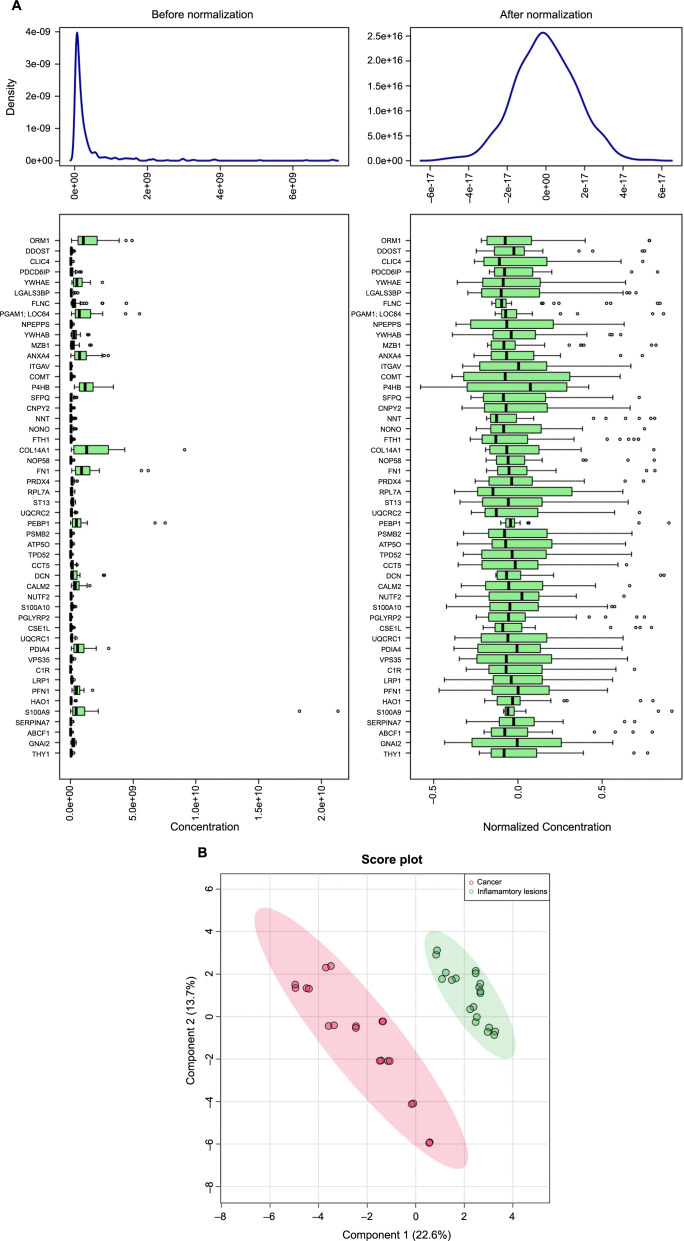
(A) Normalization of differentially expressed proteins. The left panel represents the pre-normalization curve, and the right panel represents the post-normalization curve. (B) 2D Score plot differentiating gallbladder inflammatory lesions from cancer. Principal component analysis (PCA): principal component 1 (PC1) and principal component 2 (PC2) are plotted on the *x* and *y* axes, respectively, accounting for 36.3% of the variation. Pink and green ovals represent the clustering regions of cancer and inflammatory lesion groups, respectively, with a 95% confidence interval.

The LC-MS/MS raw files (*n* ═ 40) obtained from the samples were validated using X Caliber software. The quality of the data is shown by the normalization curve and the PLsDA-2D score plot (Figure S1). The raw output data revealed 5714 proteins, 31,675 peptides, 479,279 PSMs, and 1,569,659 MS/MS spectra, as analyzed through Proteome Discoverer software. A total of 3204 proteins were filtered based on the following initial parameters: (a) Master proteins and contaminants marked as false and (b) Unique peptides greater than or equal to 2. The final set of DEPs was filtered with the additional criterion of proteins found to have sufficient confidence and peak detection in every sample. Out of 621 proteins, 18 were found to be significantly differentially expressed, with a log_2_ fold change of 1 at *P* < 0.05, where 3 were upregulated and 15 were downregulated (Table S1). Of the three significant upregulated proteins (S100P, PIGR, C1QBP), two downregulated proteins (TAGLN and CNN1) were expressed at log_2_ fold change ≥2, and the remaining 13 downregulated proteins were expressed at log_2_ fold change ≤2, as represented in the volcano plot (Figure 2).

###  PPI and pathway analysis

**Figure 3.**
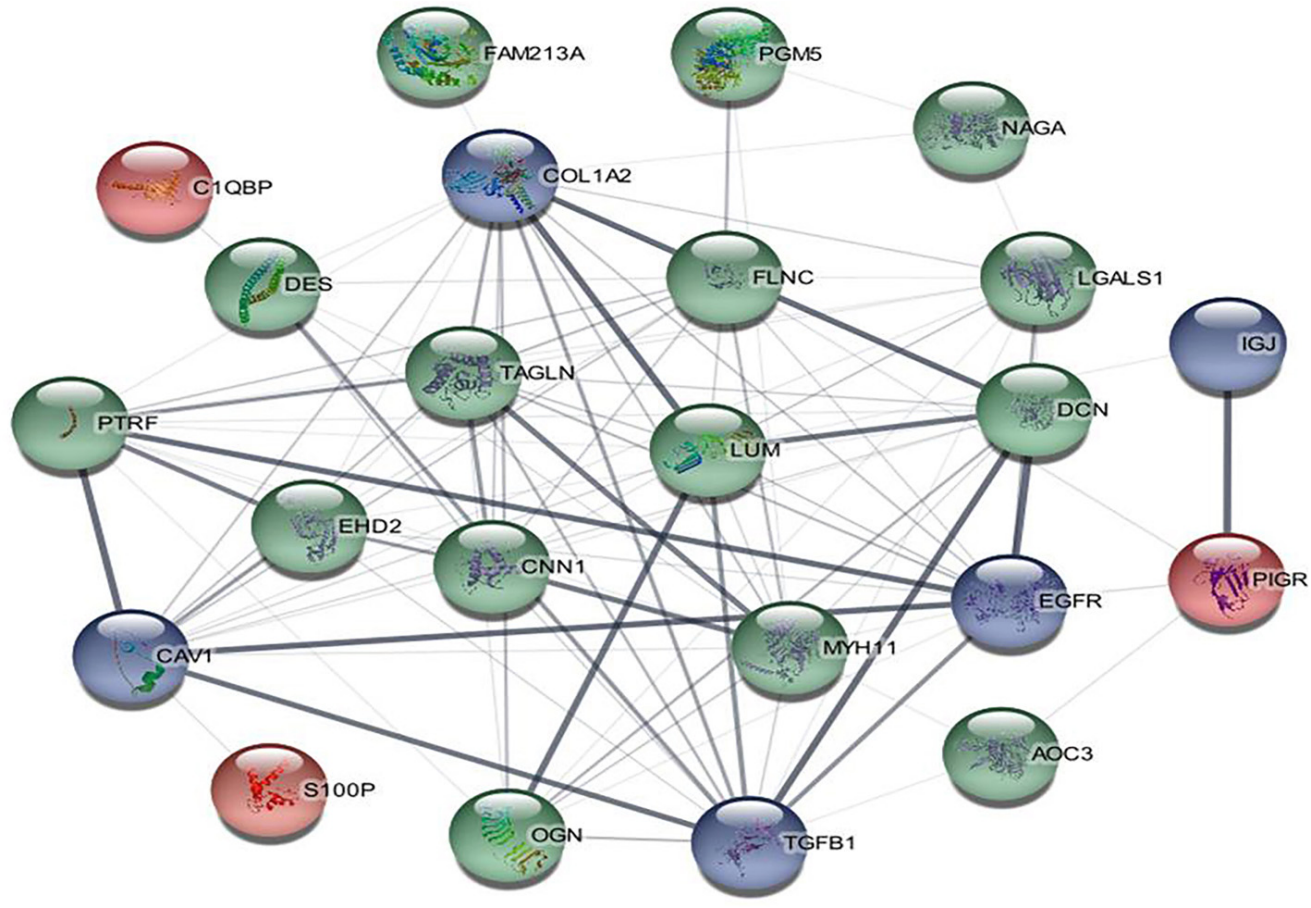
**PPI network of all 18 DEPs**. Red-colored nodes represent up-regulated proteins (S100P, PIGR, C1QBP), green-colored nodes represent downregulated proteins (TAGLN, CNN1, PTRF, DES, EHD2, PGM5, AOC3, NAGA, LUM, FLNC, DCN, MYH11, OGN, FAM213A, LGALS1) and blue-colored nodes represent other common interacting proteins (CAV1, TGFB1, EGFR, IGJ). Thick edges in between the nodes indicate high confidence in data support. S100P: S100 calcium-binding protein P; PIGR: Polymeric immunoglobulin receptor; C1QBP: Complement C1q-binding protein; TAGLN: Transgelin; CNN1: Calponin 1; PPI: Protein–protein interaction; DEPs: Differentially expressed proteins.

**Figure 4.**
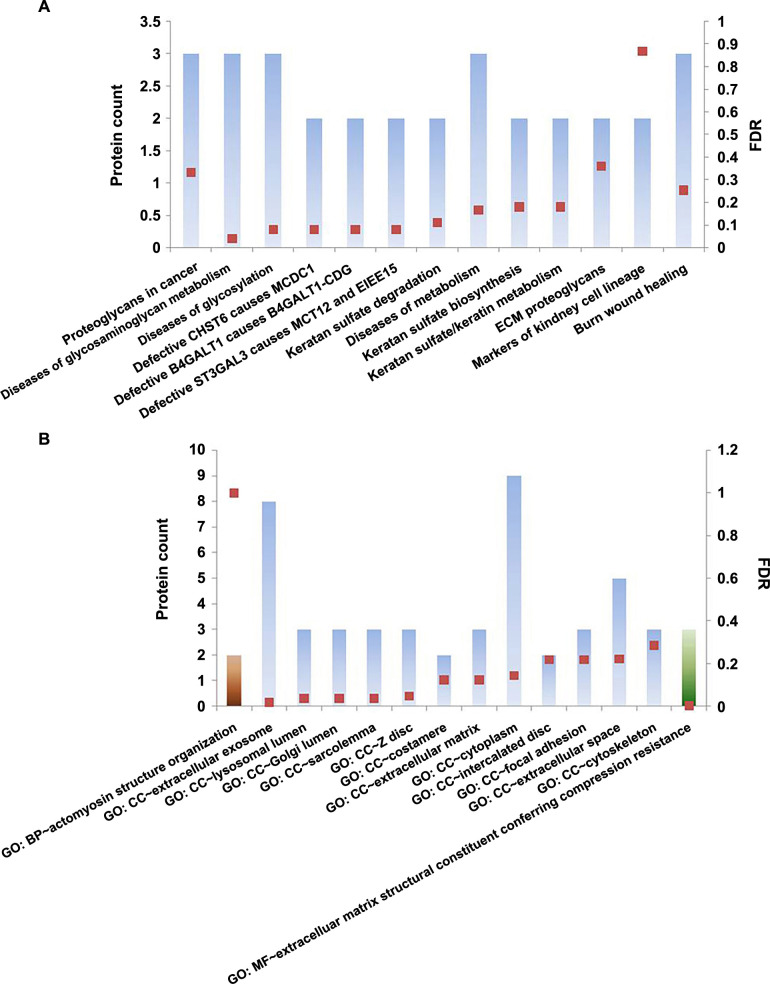
(A) Pathway analysis of DEPs through KEGG, Reactome, and Wiki pathway databases; (B) Gene Ontologies of DEPs, where the brown bar represents GO_BP, the blue bar represents GO_CC, and the green bar represents GO_MF. The square boxes designate the FDR-corrected *P* values.

The PPI network of the 18 DEPs is shown in Figure 3. TAGLN and CNN1 were identified as the two most significant nodes in the PPI network with the highest scores through cytoHubba in the majority of the topological analysis methods (Table S2). A total of 15 pathways were associated with DEPs, as shown in Figure 4A. Out of these, 13 pathways were from the Reactome database, 2 from the WikiPathways database, and 1 from the KEGG database. The KEGG database highlighted the role of DEPs in cancer-related proteoglycan pathways. The top pathways in the Reactome database were associated with glycosaminoglycan metabolism, glycosylation, and related metabolic processes. The WikiPathways database indicated that DEPs play a major role in the burn wound healing pathway. Gene Ontology for Cellular Component (GO_CC) enrichment showed that most proteins were located in the cytoplasm and extracellular exosome (Figure 4B). The extracellular space was the next most enriched cellular component. Gene Ontology for Biological Process (GO_BP) and Molecular Function (GO_MF) analysis revealed that enriched proteins were involved in actomyosin structure organization and extracellular matrix structural constituents that confer compression resistance, respectively.

### Validation by ELISA

**Figure 5.**
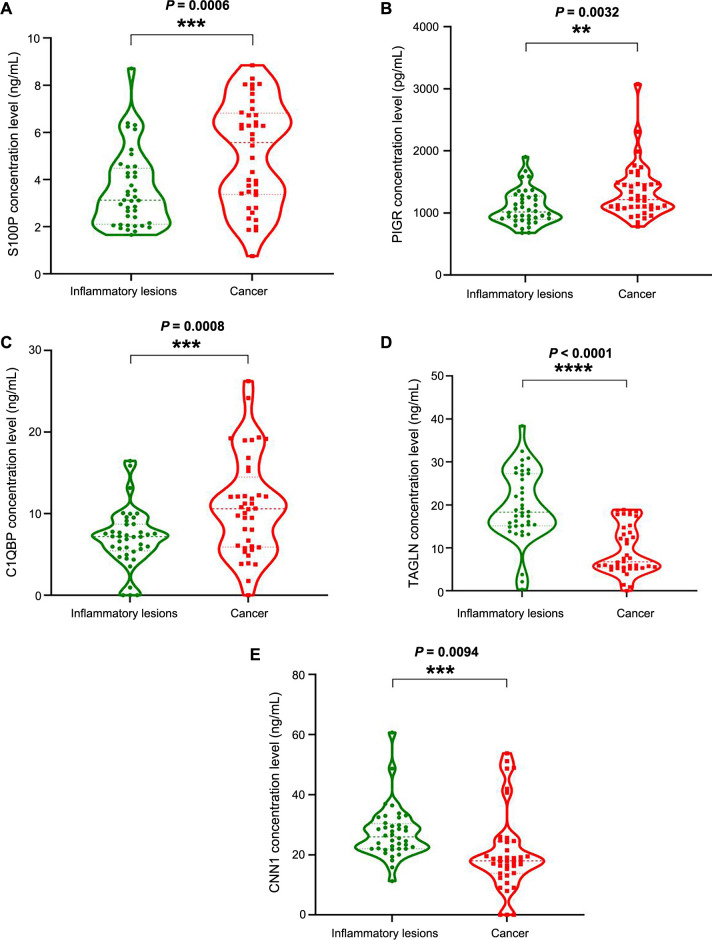
**Violin box plots showing concentration levels in inflammatory and cancer cases**. (A) S100P concentration (ng/mL) (*P* ═ 0.0006); (B) PIGR concentration (pg/mL) (*P* ═ 0.0032); (C) C1QBP concentration (ng/mL) (*P* ═ 0.0008); (D) TAGLN concentration (ng/mL) (*P* < 0.0001); (E) CNN1 concentration (ng/mL) (*P* ═ 0.0094). Each green dot represents the concentration level in individual inflammatory gallbladder cases, and each red dot represents the concentration level in individual GBC cases. S100P: S100 calcium-binding protein P; PIGR: Polymeric immunoglobulin receptor; C1QBP: Complement C1q-binding protein; TAGLN: Transgelin; CNN1: Calponin 1.

The DEPs with a log_2_ fold change ≥2 were further validated, including three upregulated proteins (S100P, PIGR, C1QBP) and two downregulated proteins (TAGLN, CNN1). A total of 80 independent technical duplicate samples (40 cancer cases and 40 inflammatory lesions) were used for validation. The staging of all 40 GBC cases used in the validation study is provided in Table S3. Protein concentration levels were measured by ELISA in tissue lysates. The mean values of S100P, PIGR, C1QBP, TAGLN, and CNN1 in GBC cases were 5.097 ± 0.3496 ng/mL, 1334 ± 67.55 pg/mL, 10.89 ± 0.9557 ng/mL, 9.377 ± 0.8957 ng/mL, and 20.63 ± 2.082 ng/mL, respectively. In the inflammatory lesions, te levels were 3.546 ± 0.2538 ng/mL, 1089 ± 43.91 pg/mL, 7.010 ± 0.5599 ng/mL, 19.70 ± 1.294 ng/mL, and 27.24 ± 1.353 ng/mL, respectively. Significant differences were observed in protein concentration levels between cancer and inflammatory lesions for S100P, PIGR, C1QBP, TAGLN, and CNN1 (*P* ═ 0.0006, *P* ═ 0.0032, *P* ═ 0.0008, *P* < 0.0001, *P* ═ 0.0094, respectively) as shown in (Figure 5).

**Figure 6.**
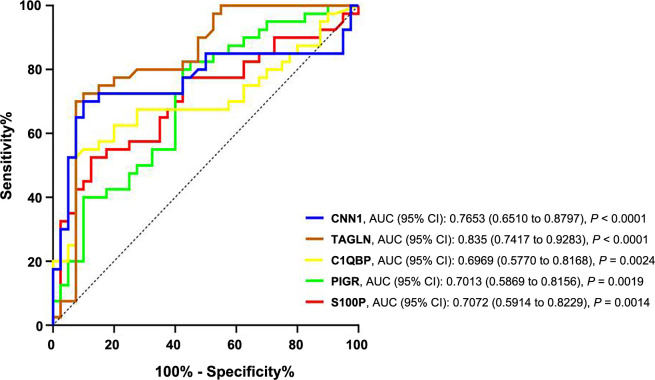
**ROC curves of S100P (red), PIGR (green), C1QBP (yellow), TAGLN (brown), and CNN1 (blue).** S100P: S100 calcium-binding protein P; PIGR: Polymeric immunoglobulin receptor; C1QBP: Complement C1q-binding protein; TAGLN: Transgelin; CNN1: Calponin 1; ROC: Receiver operating characteristic.

The receiver operating characteristic (ROC) curves were established for the signature proteins, and the area under the curve (AUC) [95% confidence interval (CI)] was statistically significant (Figure 6). The optimal cut-off values (sensitivity and specificity) for each signature protein, as determined by the Youden index, were: S100P: 5.35 ng/µg (52.5% and 87.5%), PIGR: 1068 pg/µg (80%, 57.5%), C1QBP: 10.32 (52.5% and 92.5%), TAGLN: 12.66 ng/µg (70% and 92.5%) and CNN1: 19.79 ng/µg (70% and 90%). These values were further used for clinicopathological correlation (Table 1).

### Association of DEPs with OS in GBC cases

**Figure 7.**
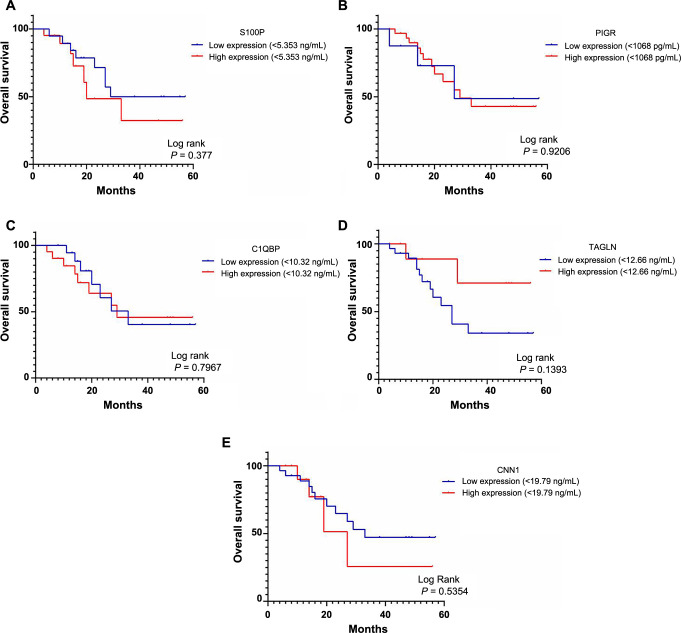
**Kaplan–Meier survival curves.** (A) S100P concentration (cut-off value 5.353 ng/mL, *P* ═ 0.377); (B) PIGR concentration (cut-off value 1068 pg/mL, *P* ═ 0.9206); (C) C1QBP concentration (cut-off value 10.32 ng/mL, *P* ═ 0.7967); (D) TAGLN concentration (cut-off value 12.66 ng/mL, *P* ═ 0.1393); (E) CNN1 concentration (cut-off value 19.79, *P* ═ 0.5354). S100P: S100 calcium-binding protein P; PIGR: Polymeric immunoglobulin receptor; C1QBP: Complement C1q-binding protein; TAGLN: Transgelin; CNN1: Calponin 1.

Survival curves were examined using the Kaplan–Meier method among 40 GBC cases, comparing the concentrations of S100P, PIGR, C1QBP, TAGLN, and CNN1 through log-rank testing. OS was defined as the time from treatment initiation to death, with follow-up concluding on February 28, 2023, and a median follow-up duration of 16 months for OS. An optimal concentration threshold for the five DEPs was used to predict OS based on high/low expression levels. It was observed that high expression of DEPs in cancer cases was associated with relatively poor OS compared to low expression levels (Figure 7). However, the protein concentrations of S100P, PIGR, C1QBP, TAGLN, and CNN1 showed no statistical significance for OS (*P* ═ 0.377, *P* ═ 0.9206, *P* ═ 0.7967, *P* ═ 0.1393, *P* ═ 0.5354, respectively).

### Analysis of prognostic factors in GBC cases using univariate and multivariate approaches

Prognostic factors were analyzed using univariate and multivariate methods to predict OS, as detailed in Table 2. Univariate analysis showed that factors, such as tumor size (T1/T2 vs T3/T4), lymph node metastasis (N0 vs N1/N2), presence of distant metastasis, total bilirubin levels (mg/dL), and unconjugated bilirubin levels (mg/dL) were significantly associated with poor OS (*P* ═ 0.005, *P* ═ 0.0001, *P* ═ 0.001, *P* ═ 0.006, *P* ═ 0.04, respectively). Other prognostic factors did not show significant correlations with OS. In multivariate Cox regression analysis, lymph node metastasis was significantly associated with poor OS (*P* ═ 0.004). In our multivariate analysis, one downregulated protein, TAGLN, was significant (*P* ═ 0.049) when all five biomarkers were evaluated together. However, when upregulated (S100P, PIGR, C1QBP) and downregulated (TAGLN, CNN1) proteins were analyzed separately, neither group significantly impacted OS, as shown in Table S5.

### Relative mRNA expression level of DEPs in GBC and inflammatory lesions

**Figure 8.**
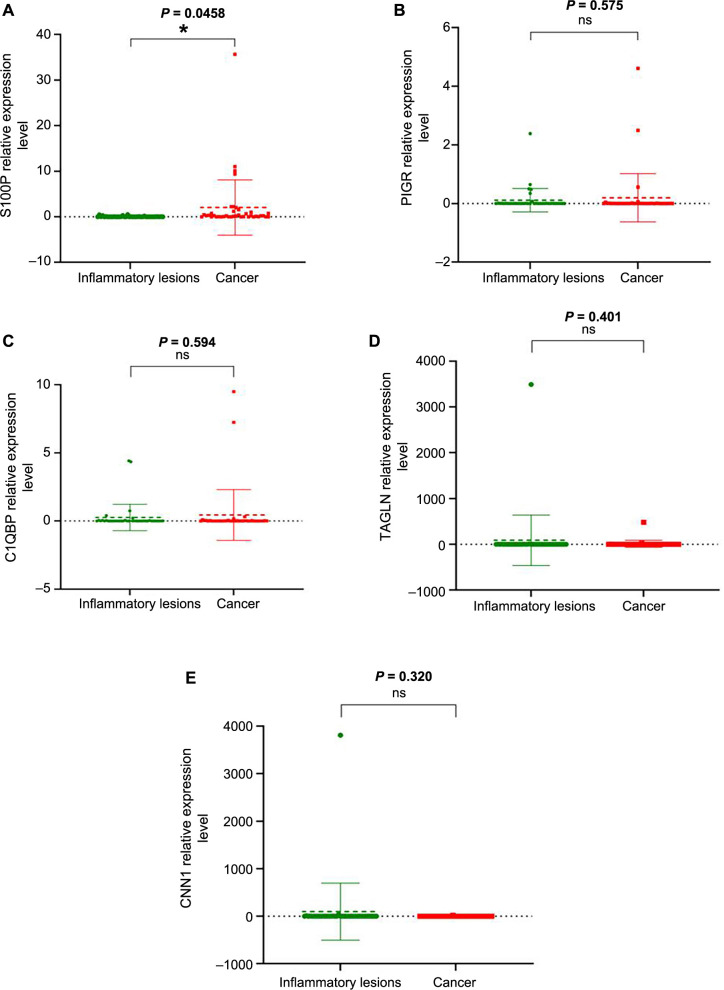
**Box violin plots showing relative mRNA expression levels in inflammatory and cancer cases.** (A) S100P (*P* ═ 0.0458); (B) PIGR (*P* ═ 0.575); (C) C1QBP (*P* ═ 0.594); (D) TAGLN (*P* ═ 0.401); (E) CNN1 (*P* ═ 0.320). Each green dot represents the relative mRNA expression level in individual inflammatory gallbladder cases, and each red dot represents the relative mRNA expression level in individual GBC cases. S100P: S100 calcium-binding protein P; PIGR: Polymeric immunoglobulin receptor; C1QBP: Complement C1q-binding protein; TAGLN: Transgelin; CNN1: Calponin 1.

The relative mRNA expression levels of S100P, PIGR, C1QBP, TAGLN, and CNN1 were analyzed in the matched 80 cases (40 GBC and 40 inflammatory lesions). A significant correlation was observed for S100P mRNA expression levels between cancer and inflammatory lesions (*P* ═ 0.0458). mRNA fold change levels were higher in cancer cases than in inflammatory lesions for all five genes studied. However, no significant correlation was found for PIGR, C1QBP, TAGLN, and CNN1 mRNA expression levels between cancer and inflammatory lesions (*P* ═ 0.575, *P* ═ 0.594, *P* ═ 0.401, *P* ═ 0.320, respectively), as shown in Figure 8.

## Discussion

The aggressiveness of GBC and the relative paucity of biological markers were the driving factors for this study. The focus was on protein-based markers, with the overall goal of community-based prevention and early detection of pre-existing inflammatory lesions. Our study appears to be the first comprehensive analysis of signature proteins, followed by their validation using independent sets of protein lysates alongside corresponding RNA-based expression. To date, there are only two previously published studies that establish diagnostic markers for GBC by comparing protein expression in both inflammatory conditions and cancer. The first study from China [18] found that Annexin A4 was upregulated, while Hsp90B and Dync1h1 were downregulated in GBC (*n* ═ 10) compared to inflammatory lesions (*n* ═ 10), using the 2DE-MALDI-TOF technique. The second, an Indian study [19], used the LC-MS/MS (iTRAQ) technique and identified that prosaposin was upregulated and transgelin (TAGLN) was downregulated in GBC (*n* ═ 10) compared to normal gallbladder tissue (*n* ═ 10). However, both studies had the following limitations: (a) the study by Huang et al. [18] was limited to the discovery phase only, with no further clinical validation; (b) clinical phenotypes of the samples used in the studies were not homogeneous; (c) findings were not clinically correlated in the studies; and (d) protein–protein interaction analysis and the functional role of identified proteins in cancer were not established.

**Table 1:** Patients’ clinicopathological characteristics correlated with concentrations of S100P, PIGR, C1QBP, TAGLN, and CNN1 at their respective optimal cut-off values (5.353 ng/µg, 1.068 ng/µg, 10.32 ng/µg, 12.66 ng/µg, and 19.79 ng/µg, respectively) (*n* ═ 80)

**Characteristics**		**Frequency (*n* ═ 80)**	**S1OOP**		***P***	**PIGR**		***P***	**C1QBP**		***P***	**TAGLN**		***P***	**CNN1**		***P***
			**Low expression (<5.35 ng/µg)**	**High expression (≥5.35 ng/µg)**		**Low expression (<1068 pg/µg)**	**High expression (≥1068 pg/µg)**		**Low expression (<10.32 ng/µg)**	**High expression (≥10.32 ng/µg)**		**Low expression (<12.66 ng/µg)**	**High expression (≥12.66 ng/µg)**		**Low expression (<19.79 ng/µg)**	**High expression (≥19.79 ng/µg)**	
**Median age (years): 52**				
Phenotype	Cancer	40 (50%)	19	21	0.0001^*^	8	32	0.0006^*^	19	21	<0.0001^*^	28	12	<0.0001^*^	28	12	<0.0001^*^
	Inflammatory lesions	40 (50%)	35	5		23	17		37	3		3	37		4	36	
Gallstone	Present	12 (15%)	9	3	0.54	7	5	0.13	12	0	0.014^*^	3	9	0.31	5	7	0.9
	Absent	68 (85%)	45	23		24	44		44	24		28	41		27	41	
Lymph node metastasis	N0	29 (72.5%)	16	13	0.24	7	22	0.55	13	16	0.001^*^	19	10	0.55	20	9	0.80
	N1	10 (25%)	3	7		1	9		42	8		8	2		7	3	
	N2	1 (2.5%)	0	1		0	1		1	0		1	0		1	0	
Differentiation	Well/ moderate	29 (72.5%)	11	18	0.049^*^	6	23	0.86	11	18	0.049^*^	21	11	0.23	19	10	0.32
	Poor	11 (27.5%)	8	3		2	9		8	3		7	1		9	2	
Tumor size	T1	9 (11.25%)	3	6	0.16	2	7	0.91	2	7	0.27	4	5	0.24	4	5	0.15
	T2	14 (17.5%)	10	4		3	11		9	5		11	3		12	2	
	T3	15 (18.75%)	5	10		3	12		7	8		11	4		10	5	
	T4	2 (2.5%)	1	1		0	2		1	1		2	0		2	0	
Metastasis	Yes	19 (47.50%)	8	11	0.52	3	16	0.53	11	8	0.21	13	4	0.44	14	5	0.63
	No	21 (52.5%)	11	10		5	16		8	13		15	8		14	7	
**Preoperative laboratory values**																	
Bilirubin (Total), mg/dL	<0.20	4 (5%)	2	2	0.74	2	2	0.08	2	2	0.63	1	3	0.76	0	4	0.10
	0.20–1.20	64 (80%)	44	20		21	43		46	18		26	38		29	35	
	>1.20	12 (15%)	8	4		8	4		8	4		4	8		3	9	
Bilirubin (conjugated), mg/dL	<0.10	5 (6.25%)	1	4	0.02^*^	1	4	0.32	1	4	0.04^*^	2	3	0.66	2	3	0.96
	0.10-0.30	51 (63.75%)	39	12		18	33		37	14		20	21		21	30	
	>0.30	24 (30%)	14	10		12	12		18	6		9	15		9	15	
Bilirubin (unconjugated), mg/dL	<0.20	10 (12.5%)	5	5	0.15	3	7	0.53	7	3	0.04^*^	6	4	0.003^*^	8	2	0.01^*^
	0.20–0.90	65 (81.25%)	47	18		25	40		48	17		20	45		21	44	
	>0.90	5 (6.25%)	2	3		3	2		1	4		5	0		3	2	
**Blood test markers**																	
CA125, U/mL	<35	2 (2.5%)	1	1	0.25	1	1	0.25	1	1	0.25	1	1	>0.9	2	0	0.25
	≥35	2 (2.5%)	0	2		2	0		0	2		1	1		1	1	
	NT	76 (95%)	53	23		28	48		55	21		29	47		29	47	
CA19.9, U/mL	<37	41 (51.25%)	30	11	0.12	15	26	0.68	31	10	0.075	16	25	0.82	14	27	0.17
	≥37	19 (23.75%)	10	9		8	11		10	9		8	11		10	9	
	NT	20 (25%)	14	6		8	12		15	5		7	13		8	12	
CEA, ng/mL	<5	38 (47.5%)	28	10	0.04^*^	17	21	0.15	26	12	0.09	13	25	0.14	11	27	0.003^*^
	≥5	10 (12.5%)	4	6		2	8		4	6		6	4		8	2	
	NT	32 (40%)	22	10		12	20		26	6		12	20		13	19	

*P < 0.05; NT: Not tested; S100P: S100 calcium-binding protein P; PIGR: Polymeric immunoglobulin receptor; C1QBP: Complement C1q-binding protein; TAGLN: transgelin; CNN1: Calponin 1.

Regarding the presence of gallbladder inflammation and its link to cancer development, it is crucial to recognize that while gallstones commonly cause inflammation, they are not the sole contributor; other factors also play a role. Our focus is on chronic inflammation in various forms, including acalculous cholecystitis (inflammation without stones), inflammation due to chronic typhoid infection, and xanthogranulomatous cholecystitis. Incidental gallbladder cancer (IGBC) cases, identified without suspicion of malignancy, further emphasize this point, with an incidence ranging from 0.19% to 3.3% [20]. These diverse inflammatory conditions, regardless of the presence of stones, are essential in understanding GBC pathogenesis.

In our study, we used a label-free LC-MS/MS technique on 20 homogeneous tissue samples (ten cancer cases and ten inflammatory lesions) for the discovery phase. At a cutoff of log_2_ fold change ≥2, we identified three significantly upregulated proteins (S100P, PIGR, C1QBP) and two downregulated proteins (TAGLN and CNN1). The remaining 13 proteins were identified as downregulated at log_2_ fold change ≤2. The five DEPs (S100P, PIGR, C1QBP, TAGLN, and CNN1) were validated by ELISA in an independent set of 80 gallbladder tissue samples (40 cancer cases and 40 inflammatory lesions). Significant differences were observed in the protein concentration levels of S100P, PIGR, C1QBP, TAGLN, and CNN1 between cancer and inflammatory lesions (*P* ═ 0.0006, *P* ═ 0.0032, *P* ═ 0.0008, *P* < 0.0001, *P* ═ 0.0094, respectively). Exposure to carcinogens can potentially transform normal gallbladder epithelium into a state of metaplasia, leading to dysplasia and eventually CIS, which can progress to invasive carcinoma. Notably, over 90% of individuals diagnosed with gallbladder carcinoma exhibit signs of dysplasia and CIS [21]. In our study, the low expression of upregulated proteins in inflammatory lesions suggests an ongoing neoplastic process beyond the morphological detection limits of available modalities.

To investigate the transcriptional profile of the signature proteins (S100P, PIGR, C1QBP, TAGLN, and CNN1) in carcinogenesis, we examined their corresponding mRNA expression in both clinical phenotypes (GBC and inflammatory lesions). The primary objective was to identify the corresponding fold changes between mRNA and protein expression levels. Although a comparable difference was noted between the clinical phenotypes at the mRNA expression level for all the genes, the majority of the samples showed negligible fold changes, which may or may not be detectable by conventional real-time PCR. Therefore, our results confirmed that a protein-based assay has relatively higher resolution for detecting changes in protein expression compared to mRNA expression and may assist in risk stratification with greater precision.

**Table 2:** Univariate and multivariate analysis of prognostic factors in GBC cases (*n* ═ 40)

**Characteristics**		***n***	**Univariate cox regression**		**Multivariate cox regression**	
			**Hazard ratio [95% CI]**	***P* value**	**Hazard ratio [95% CI]**	***P* value**
S100P	<5.35 ng/µg (ref)	19	1.58 [0.56–4.46]	0.39	0.12 [0.01–2.66]	0.18
	≥5.35 ng/µg	21				
PIGR	<1068 pg/µg (ref)	8	0.94 [0.26–3.33]	0.92	0.22 [0.01–4.87]	0.34
	≥1068 pg/µg	32				
C1QBP	<10.32 ng/µg (ref)	19	1.14 [0.41–3.15]	0.8	4.87 [0.12–7.39]	0.94
	≥10.32 ng/µg	21				
TAGLN	<12.66 ng/µg (ref)	28	0.34 [0.08–1.51]	0.16	0.73 [0.03–16.89]	0.85
	≥12.66 ng/µg	12				
CNN1	<19.79 ng/µg (ref)	28	1.38 [0.43–4.44]	0.59	3.07 [0.44–21.15]	0.26
	≥19.79 ng/µg	12				
Age	<60 (ref)	32	1.71 [0.58–5.0]	0.33	1.24 [0.08–19.08]	0.88
	≥60	8				
Gender	Male (ref)	13	0.40 [0.14–1.16]	0.09	3.52 [0.21–59.41]	0.38
	Female	27				
Tumor size	T1/T2 (ref)	23	4.89 [1.62–14.77]	0.005*	34.22 [0.60–1959.46]	0.09
	T3/T4	17				
Lymph node metastasis	N0 (ref)	29	0.05 [0.01–0.24]	0.0001*	0.01 [0.001–0.22]	0.004*
	N1/N2	11				
Differentiation	Poor (ref)	8	1.31 [0.42–4.2]	0.64		
	Well/moderate	11				
Distant Metastasis	No (ref)	21	13.04 [2.77–61.37]	0.001*	2.76 [0.07–107.49]	0.59
	Yes	19				
Gall stones	No (ref)	37	NE			
	Yes	3				
Dietary habit	Veg (ref)	24	0.78 [0.25–2.47]	0.67		
	Non veg	16				
Smoking	No (ref)	37	0.85 [0.11–6.45]	0.87		
	Yes	3				
Alcohol	No (ref)	37	1.19 [0.27–5.3]	0.82		
	Yes	3				
Urea (mg/dL)	<40.0 (ref)	36	1.33 [0.30–5.91]	0.79		
	≥40.0	4				
Creatine (mg/dL)	<1.0 (ref)	37	1.82 [0.41–8.08]	0.43		
	≥1.0	3				
Calcium (mg/dL)	<10.5 (ref)	37	1.02 [0.13–7.87]	0.98		
	≥10.5	3				
Bilirubin (Total); mg/dL	<1.20 (ref)	35	4.89 [1.58–15.11]	0.006*	9.88 [0.21–462.90]	0.24
	≥1.20	5				
Bilirubin (conjugated); mg/dL	<0.30 (ref)	29	2.32 [0.73–7.35]	0.15		
	≥0.30	11				
Bilirubin (unconjugated); mg/dL	<0.90 (ref)	35	3.39 [1.04–11.09]	0.04*	0.49 [0.06–4.24]	0.52
	≥0.90	5				
SGOT (AST); U/L	<40.00 (ref)	31	1.94 [0.60–6.28]	0.27		
	≥40.00	9				
SGPT (ALT); U/L	<45.00 (ref)	28	1.07 [0.34–3.40]	0.91		
	≥45.00	12				
Total protein, g/dL	<8.00 (ref)	36	0.53 [0.07–4.03]	0.54		
	≥8.00	4				
Albumin (g/dL)	<5.00 (ref)	39	NE	**-**		
	≥5.00	1				
Globulin (g/dL)	<3.5 (ref)	36	0.57 [0.07–4.36]	0.59		
	≥3.5	4				

*P < 0.05; NE: Not estimable; CI: Confidence interval; ref: Reference value; S100P: S100 calcium-binding protein P; PIGR: Polymeric immunoglobulin receptor; C1QBP: Complement C1q-binding protein; TAGLN: Transgelin; CNN1: Calponin 1; SGOT: Serum glutamic oxaloacetic transaminase; SGPT: Serum glutamic pyruvic transaminase.

The clinicopathological characteristics of patients in the validation phase (*n* ═ 80) were correlated with S100P, PIGR, C1QBP, TAGLN, and CNN1 concentrations at their respective optimal cut-off values (5.353 ng/µg, 1068 pg/µg, 10.32 ng/µg, 12.66 ng/µg, and 19.79 ng/µg, respectively), as shown in Table 1 and Table S4. The signal transduction mechanisms of these signature proteins were established based on our clinical outcomes and in reference to previously published studies on the identified proteins.

**Figure S2.**
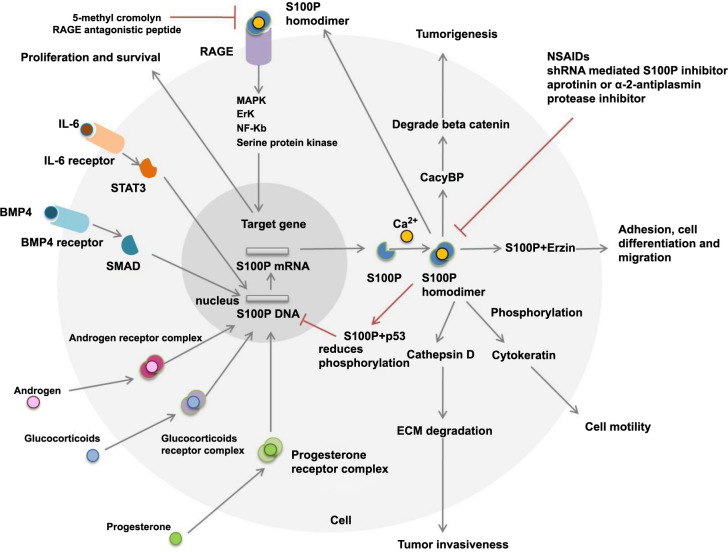
**Various signaling molecules, such as glucocorticoids, androgens, progesterone, BMP4 (which activates SMAD), and IL-6 (which activates the downstream signaling molecule STAT3), regulate the transcriptional activity of S100P in cancer cells.** The S100P protein is expressed in an inactive state and is activated by calcium ions to form active homodimers. These homodimers then interact with target proteins such as ezrin, a multidomain protein involved in adhesion, cell differentiation, and migration. Additionally, the S100P dimer activates cytokeratins through phosphorylation while reducing actin levels, ultimately affecting cell motility in cancer cells. The receptor for advanced glycation end products (RAGE), a member of the immunoglobulin superfamily, is present on the cell surface. S100P acts as a ligand that binds to RAGE, activating several signaling pathways, including mitogen-activated protein kinase (MAPK), nuclear factor-kappa B (NF-κB), extracellular signal-regulated kinase (ERK), and serine/threonine protein kinase pathways. Activation of these pathways leads to cell proliferation and survival. Another signal transduction pathway involves the interaction between CacyBP/SIP and S100P, which promotes the degradation of beta-catenin and regulates tumorigenesis in tumor cells. Moreover, the S100P homodimer upregulates the expression of cathepsin D, leading to the degradation of the extracellular matrix and promoting tumor invasiveness. A feedback loop exists in which high levels of S100P suppress endogenous S100P mRNA. The glucocorticoid-mediated S100P pathway plays a critical role in cancer therapies. During tumor progression, certain nonsteroidal anti-inflammatory drugs (NSAIDs) also impact S100P expression. In pancreatic cells, 5-methyl cromolyn, an S100P inhibitor, binds to the RAGE receptor and inhibits tumor metastasis and growth. Another therapeutic approach involves using a RAGE-antagonistic peptide that blocks the interaction between RAGE and S100P. Additionally, the invasion of S100P-positive cells is suppressed by protease inhibitors such as aprotinin or α-2-antiplasmin. S100P: S100 calcium-binding protein P.

*S100P*: This signaling molecule mediates multiple transduction pathways through Ca^2+^ ion activation, as depicted in Figure S2. In our study, S100P expression was significantly correlated with the clinical phenotype of gallbladder conditions (cancer vs inflammatory lesions; *P* ═ 0.0001), differentiation (well/moderate vs poor; *P* ═ 0.0491), calcium levels (*P* ═ 0.039), preoperative bilirubin levels (conjugated; *P* ═ 0.0189), and CEA marker (*P* ═ 0.04). These clinical findings align with our study outcomes and are consistent with established pathways in GBC. Several studies have elucidated the role of S100P as a diagnostic marker in cancers. For example, Aishima et al. [21] reported that S100P expression is associated with the progression from low-grade to high-grade biliary intraepithelial neoplasia and serves as a strong early detection marker for cholangiocarcinoma. Similarly, Mathai et al. [22] found that S100P overexpression strongly correlates with GBC advancement and poor survival. Moreover, one study suggested that LASP-1 and S100P are two therapeutic targets that inhibit GBC aggressiveness and metastasis [23].

Parkila et al. [24] evaluated S100P protein and corresponding mRNA expression levels in normal and tumor tissues of various organs using immunohistochemistry (IHC) and real-time PCR, respectively. They found that S100P protein expression was highly elevated in all tumor tissues, with the most prominent expression observed in gastric tumors. The authors [24] suggested that the high expression level of S100P in tumor tissues could serve as a potential target marker for diagnostic applications. Consistent with the above-published studies, our results also demonstrated a significant correlation between S100P mRNA expression levels in cancerous and inflammatory lesions of the gallbladder (*P* ═ 0.0458). Thus, our study indicates that high expression of S100P protein and mRNA is found in GBC compared to inflammatory lesions of the gallbladder.

**Figure S3.**
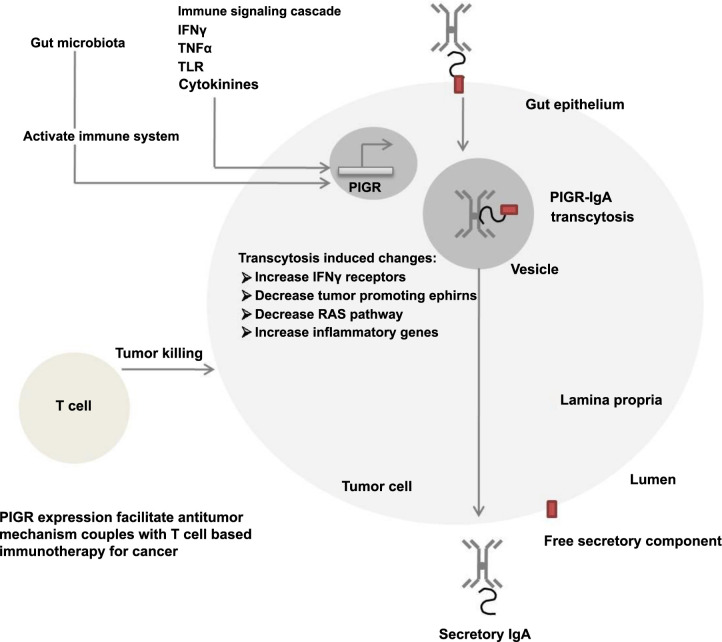
**PIGR signaling pathway in cancer.** Mechanistically, PIGR binds to dimerized IgA and IgM on the basolateral surface of gut epithelial cells, followed by the endocytosis of IgA into vesicles. PIGR then transports IgA, becoming part of the secreted IgA molecule, which is further transported from the lamina propria across the epithelial barrier to the mucosal lumen. This process enables PIGR to serve as a link between the innate and adaptive immune responses. The expression of PIGR is regulated by immune system mediators such as interferon-gamma (IFNγ) and tumor necrosis factor-alpha (TNFα). Various immune signaling cascades, including Toll-like receptor (TLR) activation and inflammatory cytokine signaling, also directly influence the upregulation of the PIGR gene. NF-κB activation subsequently enhances the transcytosis of PIGR: PIg. PIGR-IgA transcytosis induces transcriptional changes that promote inflammatory pathways in cancer cells, including the upregulation of interferon-gamma receptors, the downregulation of tumor-promoting ephrins, and antagonism of the RAS pathway. This sensitizes tumor cells to cytolytic killing by T cells. Consequently, tumor-dependent antibodies facilitate the destruction of cancer cells through antibody-dependent cellular cytotoxicity (ADCC) and antibody-dependent cellular phagocytosis (ADCP) mechanisms. PIGR: Polymeric immunoglobulin receptor.

*PIGR*: The polymeric immunoglobulin receptor (PIGR) is a transmembrane protein involved in cancer signaling pathways, as illustrated in Figure S3. In the current study, PIGR expression was significantly correlated with the clinical phenotype (cancer vs inflammatory lesions; *P* ═ 0.0006) and AFP (*P* ═ 0.045). Similar results are available in the Human Protein Atlas (HPA) database (https://www.proteinatlas.org/), which indicates high PIGR expression in cancerous tissues of the gastrointestinal mucosa, kidney, gallbladder, and urinary bladder compared to non-cancerous tissues. Increased PIGR expression has also been observed in the gastrointestinal tract and hepatocellular carcinoma [25]. Our study showed higher PIGR expression in GBC tissues compared to inflammatory lesions of the gallbladder, suggesting its potential as an early detection marker in pre-existing inflammatory lesions.

A previously published study by Okhuma et al. [26] compared PIGR mRNA (data downloaded from the TCGA database) and protein (IHC) expression in pancreatic cancer. It was observed that expression was higher in the treated group compared to the untreated group, further supporting that higher levels of PIGR mRNA and protein are independent prognostic factors. To the best of our knowledge, our study is the first to assess PIGR mRNA and protein levels in clinical samples. The results of our study are consistent with previously published findings.

**Figure S4.**
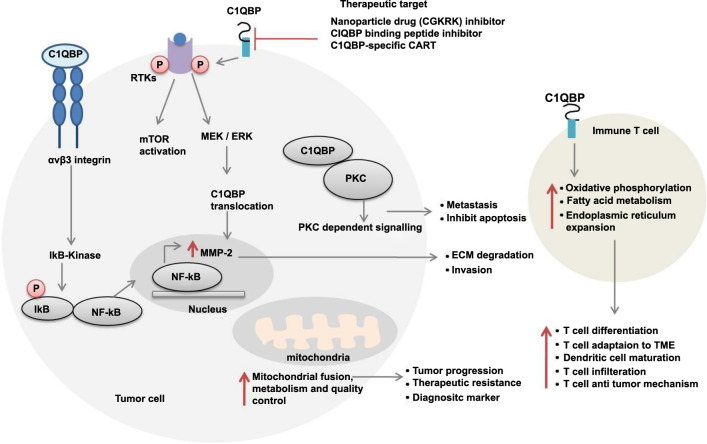
**C1QBP function as a ligand in cancer cell signaling pathway.** C1QBP binds to αvβ3 integrin and recruits IκB kinase, which phosphorylates IκB, leading to the translocation of NF-κB into the nucleus. This promotes the expression of metalloproteinases, resulting in extracellular matrix (ECM) degradation and cancer cell invasion. C1QBP also phosphorylates receptor tyrosine kinases, activating the mTOR and MEK/ERK pathways. ERK facilitates the translocation of C1QBP to the nucleus, where it binds to PKC, activating PKC-dependent signaling, which is crucial for metastasis and the inhibition of apoptosis. In mitochondria, C1QBP enhances mitochondrial fusion, metabolism, and quality control, promoting tumor progression, therapeutic resistance, and serving as a diagnostic marker. In immune cells, C1QBP increases oxidative phosphorylation, fatty acid metabolism, and endoplasmic reticulum expansion. These processes contribute to T cell differentiation, adaptation to the tumor microenvironment (TME), dendritic cell maturation, T cell infiltration, and antitumor mechanisms. Targeting C1QBP through CAR T cell therapy is a promising approach for cancer treatment. Additionally, nanoparticle drugs such as CGKRK and C1QBP-binding peptide inhibitors are potential therapeutic targets. C1QBP: Complement C1q-binding protein.

*C1QBP*: Complement C1q binding protein is involved in cancer signaling pathways, as depicted in Figure S4. Our study revealed significantly higher expression of C1QBP in GBC compared to inflammatory lesions (*P* < 0.0001). Previous studies [27–30] have suggested that C1QBP acts as a diagnostic marker in cancer patients and is related to metastasis, progression, and poor OS. Our study on GBC aligns with these established findings, reporting a significant correlation between C1QBP and lymph node metastasis (*P* ═ 0.0010), tumor differentiation (well/moderate vs poor; *P* ═ 0.0491), cholelithiasis (*P* ═ 0.0139), and preoperative bilirubin levels (conjugated: *P* ═ 0.0408; unconjugated: *P* ═ 0.0406).

In a study by Shen et al. [31], C1QBP mRNA and protein expression were compared in cholangiocarcinoma cell lines (four cell lines) and normal cell lines (one cell line) using RT-PCR and western blot, respectively. They found significantly higher C1QBP mRNA and protein expression in the cholangiocarcinoma cell lines compared to the normal cell lines. Our study is unique, as there is no published data available to date that reports C1QBP protein and mRNA expression in tissue lysates from GBC cases.

**Figure S5.**
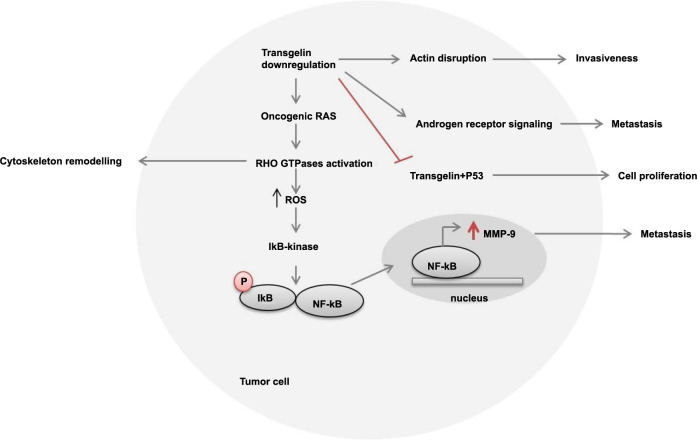
**TAGLN signaling pathway involved in cancer.** TAGLN functions as a tumor suppressor gene and is found to be downregulated in tumor cells. The downregulation of TAGLN leads to actin disruption, resulting in increased cell invasiveness. In the signal transduction pathway, decreased TAGLN expression activates the oncogenic RAS pathway, which in turn activates RHO GTPases, promoting cytoskeleton remodeling and increasing reactive oxygen species (ROS) levels. Subsequently, IκB kinase is recruited, phosphorylating IκB and translocating NF-κB to the nucleus. This triggers the overexpression of metallomatrix protease-9 (MMP9), which drives metastasis. Additionally, the reduced expression of TAGLN activates androgen receptor signaling, further contributing to metastasis. Moreover, low TAGLN expression inhibits its binding to p53, leading to unchecked cell proliferation. TAGLN: Transgelin.

*TAGLN*: Transgelin is an actin-binding protein that serves as a marker for smooth muscle differentiation [32]. The gradual loss of TAGLN function contributes to tumor progression and serves as a diagnostic marker in breast and colon cancer development [33, 34]. Our study also observed a similar trend, reporting lower expression of TAGLN in GBC compared to inflammatory lesions (*P* < 0.0001). Additionally, we found that TAGLN depletion inversely correlates with preoperative bilirubin levels (unconjugated; *P* ═ 0.003), which serves as an indicative marker of tumorigenicity in cancer cells. The signal transduction pathway involved in cancer cells is illustrated in Figure S5.

A study by Tsui et al. [35] compared the expression levels of TAGLN in bladder carcinoma cells to normal bladder tissues using RT-PCR and western blot analysis. They observed higher mRNA and protein expression levels of TAGLN in normal tissues compared to carcinoma cells. In our study, we found that the fold change level of mRNA in inflammatory lesions was relatively higher compared to the fold change level of mRNA expression in cancer cases. However, no significant correlation was found.

**Figure S6.**
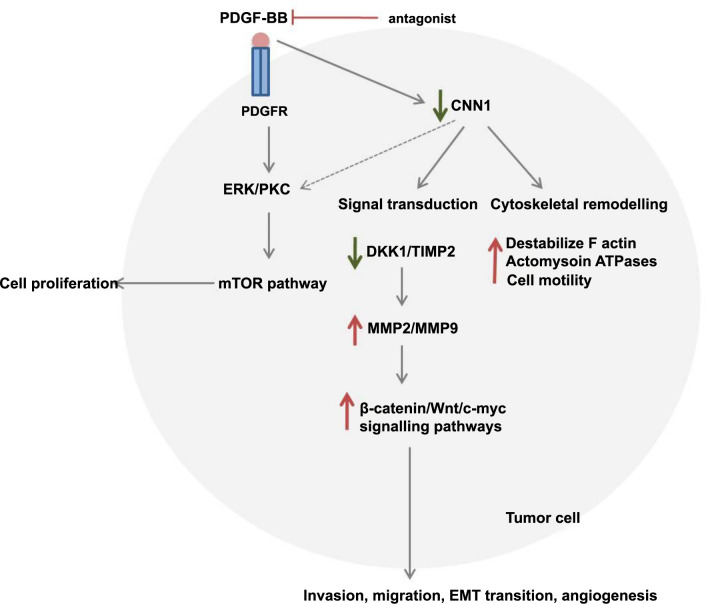
**CNN1 signaling pathway involved in cancer.** CNN1 recruits PKC/ERK through its structural domain, further activating the mTOR pathway, which leads to cell proliferation. Malignant tumor cells secrete various growth factors, such as PDGF-BB, which suppress CNN1 expression. CNN1 plays a crucial role in cytoskeletal remodeling and signal transduction due to its structural domain. Decreased CNN1 expression destabilizes F-actin, enhancing cell motility. In signal transduction, low CNN1 expression reduces levels of dickkopf-1 (DKK1) and tissue inhibitor of metalloproteinases-2 (TIMP-2). This leads to the overexpression of metalloproteinases (MMP2/MMP9), activating the β-catenin/Wnt/c-myc signaling pathways, which promote cell invasion, migration, epithelial–mesenchymal transition (EMT), and angiogenesis in tumor cells. A potential therapeutic approach involves the use of PDGF antagonists, such as neomycin, to target these pathways in cancer treatment. CNN1: Calponin 1.

*CNN1*: The calponin protein plays a crucial role as a cytoskeletal protein and mediator of signal transduction, as illustrated in Figure S6. Several studies have identified CNN1 as a tumor suppressor, noting its decreased expression in various cancers, such as ovarian cancer, hepatocellular carcinoma, breast cancer, and colorectal cancer (CRC) [36–39]. Consistent with these findings, we also observed low expression of CNN1 in GBC compared to inflammatory lesions. Furthermore, our findings showed significant correlations between CNN1 expression levels and clinical phenotypes (cancer vs inflammatory lesions; *P* < 0.0001), urea (*P* ═ 0.0077), preoperative bilirubin levels (unconjugated; *P* ═ 0.0106), globulin (*P* ═ 0.0001), CEA (*P* ═ 0.003), and AFP (*P* ═ 0.05), suggesting its involvement in cell invasion and progression through pre-existing inflammatory conditions. A previously published study by Mamoor et al. [38] elucidated the role of CNN1 mRNA and protein expression in breast cancer tissues and adjacent normal tissues using a dataset from GEO2R. They reported decreased CNN1 expression in tumor tissues compared to adjacent normal tissues, correlating with poor OS in patients.

Our study is likely the first to report TAGLN and CNN1 protein expression and their corresponding mRNA expression in tissue lysates of GBC cases and inflammatory lesions.

As per Kaplan–Meier curve analysis, high S100P, PIGR, C1QBP, TAGLN, and CNN1 protein concentrations in GBC cases resulted in poor OS (*P* ═ 0.377, *P* ═ 0.9206, *P* ═ 0.7967, *P* ═ 0.1393, *P* ═ 0.5354, respectively). Further univariate and multivariate analyses in GBC cases were conducted to investigate the prognostic factors involved in OS. In the univariate analysis, tumor size (T1/T2 vs T3/T4), lymph node metastasis (N0 vs N1/N2), and distant metastasis (with vs without metastasis) showed significant correlations with poor OS (*P* ═ 0.005, *P* ═ 0.0001, *P* ═ 0.001, respectively). Several studies [40–42] have also elucidated that tumor size is related to poor OS in GBC patients. Further studies [43–47] reported that lymph node metastasis and distant metastasis are critical prognostic factors. Our study results showed a significant correlation between preoperative total bilirubin levels (mg/dL) and unconjugatedbilirubin levels (mg/dL) with poor OS (*P* ═ 0.006, *P* ═ 0.04, respectively). It was also observed that the hazard ratio for tumor size increases (from 4.8 to 7.7) with elevated bilirubin (total) levels in the adjusted univariate analysis. Similar to our results, three previously published studies [48–50] reported that high preoperative bilirubin levels are independent prognostic markers for poor OS in GBC.

In the multivariate analysis, lymph node metastasis (N0 vs N1/N2) revealed a significantly worse prognosis in cancer cases (*P* ═ 0.004). Our multivariate analysis also observed a significant correlation between the five DEPs and both tumor size (*P* ═ 0.09) and lymph node metastasis (*P* ═ 0.04). These findings suggest that the five DEPs, along with other parameters, impact patient survival by increasing the hazard ratio for tumor size from 4.89 to 34.22. When all five biomarkers were evaluated together for their impact on OS, TAGLN emerged as significant (*P* ═ 0.049), indicating its potential as a prognostic marker. However, when the upregulated proteins (S100P, PIGR, C1QBP) and downregulated proteins (TAGLN, CNN1) were evaluated separately, neither group showed a significant impact on OS. Furthermore, our adjusted univariate analysis revealed that the hazard ratio for tumor size increases with bilirubin levels, rising from 4.89 to 5.7. Based on all the above results, this suggests a potential unexplored relationship between the expression levels of the DEPs and bilirubin levels, which may contribute to the progression of gallbladder inflammatory lesions to invasive cancer.

The identified signature proteins need further validation in serum/plasma for clinical diagnostic use in future large cohorts. This may establish robust diagnostic parameters that may be missed due to the tissue heterogeneity of samples and the small sample size used in the validation phase.

## Conclusion

This study identified a panel of five protein-based diagnostic biomarkers (S100P, PIGR, C1QBP, TAGLN, and CNN1) potentially involved in the progression of gallbladder inflammatory lesions to invasive cancer. The signal transduction mechanisms highlighted the roles of these signature proteins in cancer metastasis and invasiveness. However, due to the small sample size, further analysis with a larger cohort is necessary to validate these biomarkers for diagnostic purposes. The study also suggests that protein-based assays may provide better resolution for GBC risk stratification compared to mRNA-based assays for future clinical use.

## Supplemental data

**Table S1:** Differentially expressed proteins by LC-MS/MS at Log_2_ fold change of 1 and *P* < 0.05

**Accession**	**Gene symbol**	**Chromosome**	**Coverage [%]**	**MW [kDa]**	**calc. pI**	**PSMs**	**Unique peptides**	**Log_2_FC at *P* < 0.05**	**Abundance ratio: (Cancer) / (Inflammatory lesion (control))**
P25815	S100P	4	72	10.4	4.88	134	4	2.7	6.654
P01833	PIGR	1	61	83.2	5.74	347	32	2.1	4.569
A8K651	C1QBP	17	50	31.4	4.84	143	7	2	4.22
Q01995	TAGLN	11	67	22.6	8.84	785	14	2.1	0.219
P51911	CNN1	19	62	33.2	9.07	312	11	2	0.247
B4DPZ5	PTRF	17	26	40.5	5.25	148	8	1.8	0.269
Q53SB5	DES	2	68	53.5	5.27	990	24	1.7	0.292
Q9NZN4	EHD2	19	55	61.1	6.46	426	21	1.6	0.317
Q15124	PGM5	9	65	62.2	7.21	308	24	1.6	0.324
Q16853	AOC3	17	27	84.6	6.52	253	13	1.6	0.322
P17050	NAGA	22	21	46.5	5.19	67	6	1.6	0.313
P51884	LUM	12	41	38.4	6.61	863	8	1.3	0.392
Q14315	FLNC	7	59	290.8	5.97	1133	92	1.2	0.421
P07585	DCN	12	47	39.7	8.54	202	12	1.2	0.428
P35749	MYH11	16	56	227.2	5.5	2512	75	1.2	0.426
Q7Z532	OGN	9	44	33.9	5.48	247	11	1	0.497
Q9BRX8	FAM213A	10	21	25.7	8.84	59	4	1	0.498
A0A384MR27	LGALS1	22	90	14.7	5.5	677	9	1	0.469

LC-MS/MS: Label-free liquid chromatography assisted tandem mass spectrometry.

**Table S2:** Identification of hub genes through cytoHubba

**Closeness method**				**Degree**				**MNC**				**MCC**				**EPC**				**Bottleneck**			
**Rank**	**Gene name**	**Score**		**Rank**	**Gene name**	**Score**		**Rank**	**Gene name**	**Score**		**Rank**	**Gene name**	**Score**		**Rank**	**Gene name**	**Score**		**Rank**	**Gene name**	**Score**	
1	COL1A2	19		1	COL1A2	16		1	COL1A2	15		1	COL1A2	168003		1	COL1A2	9.546		1	COL1A2	5	
2	MYH11	18		2	MYH11	14		2	MYH11	14		2	TAGLN	168000		2	TAGLN	9.515		2	MYH11	3	
2	FLNC	18		2	FLNC	14		2	FLNC	14		3	DCN	167760		3	DCN	9.337		2	FLNC	3	
2	TAGLN	18		2	TAGLN	14		2	TAGLN	14		4	LUM	156241		4	EGFR	9.237		4	LUM	2	
5	TGFB1	17.5		5	EGFR	13		5	EGFR	13		5	CAV1	147601		5	CAV1	9.225		4	DES	2	
6	EGFR	17.33333		5	DCN	13		5	DCN	13		6	PTRF	141120		6	MYH11	9.21		4	CAV1	2	
6	DCN	17.33333		5	LUM	13		5	TGFB1	13		7	FLNC	131164		7	LUM	9.164		7	C1QBP	1	
6	LUM	17.33333		5	CAV1	13		8	LUM	12		8	EGFR	116642		8	TGFB1	9.163		7	EGFR	1	
6	CAV1	17.33333		5	TGFB1	13		8	CAV1	12		9	MYH11	101044		9	FLNC	9.056		7	DCN	1	
10	PTRF	16.83333		10	PTRF	12		8	PTRF	12		10	LGALS1	55442		10	PTRF	8.899		7	EHD2	1	
**EcCentricity**				**DMNC**				**Betweeness**				**Radiality**				**Stress**				**Clustering coefficient**		
**Rank**	**Gene name**	**Score**		**Rank**	**Gene name**	**Score**		**Rank**	**Gene name**	**Score**		**Rank**	**Gene name**	**Score**		**Rank**	**Gene name**	**Score**		**Rank**	**Gene name**	**Score**	
1	COL1A2	0.5		1	LUM	0.848828		1	COL1A2	74.30815		1	COL1A2	3.954545		1	COL1A2	158		1	OGN	0.944444	
1	MYH11	0.5		1	CAV1	0.848828		2	FLNC	44.1176		2	MYH11	3.863636		2	DES	136		2	EHD2	0.916667	
1	CNN1	0.5		3	DCN	0.843025		3	CAV1	43.8899		2	FLNC	3.863636		3	FLNC	124		3	PTRF	0.848485	
1	FLNC	0.5		4	PTRF	0.819558		4	DES	42.18182		2	TAGLN	3.863636		4	MYH11	110		4	DCN	0.846154	
1	TAGLN	0.5		5	OGN	0.811459		5	MYH11	41.04856		5	TGFB1	3.818182		5	CAV1	104		5	CNN1	0.836364	
1	TGFB1	0.5		6	TAGLN	0.799541		6	LUM	36.39206		6	EGFR	3.772727		6	LUM	96		6	TAGLN	0.78022	
7	EGFR	0.333333		7	EHD2	0.787593		7	TGFB11	26.09452		6	DCN	3.772727		7	TGFB1	90		7	LGALS1	0.763636	
7	DCN	0.333333		8	CNN1	0.780531		8	EGFR	16.78283		6	LUM	3.772727		8	TAGLN	68		8	EGFR	0.74359	
7	EHD2	0.333333		9	EGFR	0.74084		9	LGALS1	13.02475		6	CAV1	3.772727		9	EGFR	54		8	LUM	0.74359	
7	OGN	0.333333		10	COL1A2	0.72107		10	TAGLN	10.84149		10	CNN1	3.727273		10	CNN1	38		8	CAV1	0.74359	

**Table S3:** Clinical staging characteristics of patients included in the validation phase of the study (*n* ═ 40)

**S. No.**	**Clinical sample**	**Phenotype**	**Staging**
1	Sample 1	Cancer	pT2N0
2	Sample 2	Cancer	pT2N2
3	Sample 3	Cancer	pT2aN0
4	Sample 4	Cancer	pT3pNo
5	Sample 5	Cancer	pT1bpN0
6	Sample 6	Cancer	pT2apN0
7	Sample 7	Cancer	pT2b pN0 cM0
8	Sample 8	Cancer	pT4N1M1
9	Sample 9	Cancer	pT2apN1cM0
10	Sample 10	Cancer	pT3pN1cM0
11	Sample 11	Cancer	pT3N0Mx
12	Sample 12	Cancer	pT2bN0
13	Sample 13	Cancer	pT2a
14	Sample 14	Cancer	pT3 No
15	Sample 15	Cancer	pT3pN0
16	Sample 16	Cancer	pT3pN0cM0
17	Sample 17	Cancer	pT3 pN0
18	Sample 18	Cancer	pT1b pNo
19	Sample 19	Cancer	pT3pN0
20	Sample 20	Cancer	pT3pN1
21	Sample 21	Cancer	pT2pN0Mx
22	Sample 22	Cancer	ypT3N0Mx
23	Sample 23	Cancer	pT2 pN0 cM0
24	Sample 24	Cancer	pT2apN1
25	Sample 25	Cancer	pT1bpN0
26	Sample 26	Cancer	pT3pN0
27	Sample 27	Cancer	pT1b pNo
28	Sample 28	Cancer	pT2b pNo
29	Sample 29	Cancer	pT3N1Mx
30	Sample 30	Cancer	pT3N1
31	Sample 31	Cancer	pTis pN0 cM0
32	Sample 32	Cancer	pT3No
33	Sample 33	Cancer	pT1bpN0Mx
34	Sample 34	Cancer	T1a No
35	Sample 35	Cancer	pT3pM1
36	Sample 36	Cancer	PT1b No
37	Sample 37	Cancer	pT1b pNo
38	Sample 38	Cancer	pT2 pN1 cM0
39	Sample 39	Cancer	pT4N1M0
40	Sample 40	Cancer	pT2aN0

**Table S4:** Patients’ clinico-pathological characteristics correlated with S100P, PIGR, C1QBP, TAGLN, and CNN1 concentration at their respective optimum cut-off values (5.353 ng/µg, 1.068 ng/µg, 10.32 ng/µg, 12.66 ng/µg and 19.79 ng/µg respectively) (n ═ 80)

**Characteristics**		**Frequency** **(*n* ═ 80)**	**S1OOP**		***P***	**PIGR**		***P***	**C1QBP**		***P***	**TAGLN**		***P***	**CNN1**		***P***
			**Low expression (<5.35 ng/µg)**	**High expression (≥5.35 ng/µg)**		**Low expression (<1068 pg/µg)**	**High expression (≥1068 pg/µg)**		**Low expression (<10.32 ng/µg)**	**High expression (≥10.32 ng/µg)**		**Low expression (<12.66 ng/µg)**	**High expression (≥12.66 ng/µg)**		**Low expression (<19.79 ng/µg)**	**High expression (≥19.79 ng/µg)**	
**Median age, years: 52**																	
Age	<60	56 (70%)	37	19	0.67	18	38	0.06	38	18	0.52	24	32	0.25	25	31	0.2
	≥60	24 (30%)	17	7		13	11		18	6		7	17		7	17	
Gender	Female	42 (52.5%)	25	17	0.10	14	28	0.3	27	15	0.24	18	24	0.43	21	21	0.05
	Male	38 (47.5%)	29	9		17	21		29	9		13	25		11	27	
Smoking	Yes	5 (6.25%)	3	2	0.71	2	3	0.95	4	1	0.61	3	2	0.313	3	2	0.35
	No	75 (93.7%)	51	24		29	46		52	23		28	47		29	46	
Chewing smoking	Yes	3(3.75%)	2	1	0.97 0.71	0	3	0.16 0.95	3	0	0.25 0.61	1	2	0.84 0.313	1	2	0.81 0.35
	Yes	5 (6.25%)	3	2		2	3		4	1		3	2		3	2	
Alcohol	Yes	6 (7.5%)	4	2	0.96	2	4	0.77	4	2	0.85	3	3	0.56	3	3	0.60
	No	74 (92.5%)	50	24		29	45		52	22		28	46		29	45	
Dietary habit	Vegetarian	44 (55%)	28	16	0.41	14	30	0.16	28	16	0.17	15	29	0.34	21	23	0.12
	Non-vegetarian	36 (45%)	26	10		17	19		28	8		16	20		11	25	
**Preoperative laboratory values**																	
Urea (mg/dL) 10.00-40.00	<10.0	3 (3.7%)	2	1	0.99	1	2	0.83	3	0	0.51	1	2	0.83	1	2	0.0077^*^
	10.00-40.00	71 (88.75%)	48	23		27	44		49	22		27	44		25	46	
	>40.0	6 (7.5%)	4	2		3	3		4	2		3	3		6	0	
Creatine (mg/dL)	<0.5	12 (15%)	7	5	0.4	6	6	0.66	9	3	0.46	6	6	0.66	5	7	0.98
	0.50–1.00	56 (70%)	37	19		21	35		37	19		21	35		22	34	
	>1.00	12 (15%)	10	2		4	8		10	2		4	8		5	7	
Calcium (mg/dL)	<8.5	17 (21%)	12	5	0.04^*^	7	10	0.37	12	5	0.99	6	11	0.33	5	12	0.18
	8.50-10.50	60 (75%)	42	18		24	36		42	18		25	35		27	33	
	>10.50	3 (3.75%)	0	3		0	3		2	1		0	3		0	3	
SGOT (AST); U/L	<5.00	1 (1.25%)	0	1	0.23	0	1	0.48	0	1	0.07	0	1	0.67	1	0	0.38
	5.00-40.00	58 (72.5%)	38	20		21	37		38	20		22	36		24	34	
	>40.00	21 (26.25%)	16	5		10	11		18	3		9	12		7	14	
SGPT (ALT); U/L	<5.00	0	0	0	0.41	0	0	0.69	0	0	0.99	0	0	0.51	0	0	0.29
	5.00-45.00	60 (75%)	39	21		24	36		42	18		22	38		22	38	
	>45.00	20 (25%)	15	5		7	13		14	6		9	11		10	10	
Total protein g/dL	<6.00	5 (6.25%)	2	3	0.37	1	4	0.23	2	3	0.32	3	2	0.09	2	3	0.63
	6.00-8.00	61 (76.2%)	41	19		22	39		44	17		26	35		26	35	
	>8.00	14 (17.5%)	10	4		8	6		10	4		2	12		4	10	
Albumin g/dL	<3.50	14 (17.5%)	11	3	0.62	8	6	0.3	10	4	0.37	5	9	0.94	4	10	0.43
	3.50-5.00	63 (78.75%)	41	22		22	41		45	18		25	38		26	37	
	>5.00	3 (3.75%)	2	1		1	2		1	2		1	2		2	1	
Globulin (g/dL)	<2.00	3 (3.75%)	2	1	0.19	1	2	0.25	1	2	0.07	2	1	0.07	1	2	0.0001^*^
	2.00-3.50	59 (73.75%)	37	22		20	39		39	20		26	33		16	43	
	>3.50	18 (22.5%)	15	3		10	8		16	2		3	15		15	3	
**Blood test markers**																	
AFP(ng/mL)	<8.78	3 (3.75%)	1	2	0.25	0	3	0.045^*^	2	1	0.25	2	1	0.25	3	0	0.05^*^
	≥8.78	1 (1.25%)	1	0		1	0		0	1		0	1		0	1	
	NT	76 (95%)	52	24		30	46		54	22		29	47		29	47	

*P < 0.05; NT: Not tested; S100P: S100 calcium-binding protein P; PIGR: Polymeric immunoglobulin receptor; C1QBP: Complement C1q-binding protein; TAGLN: Transgelin; CNN1: Calponin 1.

**Table S5:** Multivariate analysis of prognostic factors in GBC cases (*n* ═ 40)

**Characteristics**		***n***	**Multivariate cox regression (S100P, PIGR, C1QBP, TAGLN, CNN1)**		**Multivariate cox regression (Upregulating proteins-S100P, PIGR, C1QBP)**		**Multivariate cox regression (Downregulating proteins-TAGLN, CNN1)**	
			**Hazard ratio [95% CI]**	***P* value**	**Hazard ratio [95% CI]**	***P* value**	**Hazard ratio [95% CI]**	***P* value**
S100P	<5.35 ng/µg (ref)	19	1.45[0.394–5.35]	0.57	1.7[0.535-5.42]	0.36	0.321[0.07-1.45]	0.14
	≥5.35 ng/µg	21						
PIGR	<1068 pg/µg (ref)	8	1.45 [0.29–7.17]	0.644	0.768 [0.198-2.97]	0.703	1.54 [0.47-5.09]	0.47
	≥1068 pg/µg	32						
C1QBP	<10.32 ng/µg (ref)	19	2.07 [0.525–8.21]	0.297	0.97 [0.33-2.84]	0.967	–	–
	≥10.32 ng/µg	21						
TAGLN	<12.66 ng/µg (ref)	28	0.17 [0.03–0.99]	0.049*	–	–	–	**–**
	≥12.66 ng/µg	12						
CNN1	<19.79 ng/µg (ref)	28	1.18 [0.32–4.23]	0.79	–	–	–	–
	≥19.79 ng/µg	12						

*P < 0.05. S100P: S100 calcium-binding protein P; PIGR: Polymeric immunoglobulin receptor; C1QBP: Complement C1q-binding protein; TAGLN: Transgelin; CNN1: Calponin 1.

## Data Availability

Data is available via Proteome X change consortium through MassIVE data repository with the identifier PXD040704/ MSV000091448**.**

## References

[1] Abou-Alfa GK, Jarnagin W, El Dika I, D’Angelica M, Lowery M, Brown K, et al.

[2] Bray F, Ferlay J, Soerjomataram I, Torre LA, Jemal A, Siege RL (2018). Global cancer statistics 2018: GLOBOCAN estimates of incidence and mortality worldwide for 36 cancers in 185 countries. CA Cancer J Clin.

[3] Schmidt MA, Marcano-Bonilla L, Roberts LR (2019). Gallbladder cancer: epidemiology and genetic risk associations. Chin. Clinl Oncol.

[4] Popli P, Kapoor VK, Dutta U, Bush N, Kalsi D (2019). Epidemiology of gallbladder cancer in India. Chin Clin Oncol.

[5] Albores-Saavedra J, Alcantra-Vazquez A, Cruz-Ortiz H, Herrera-Goepfert R R (1980). The precursor lesions of invasive gallbladder carcinoma. Hyperplasia, atypical hyperplasia and carcinoma in situ. Cancer.

[6] Roa I, Araya JC, Villaseca M, De Aretxabala X, Riedemann P, Endoh K (1996). Preneoplastic lesions and gallbladder cancer: an estimate of the period required for progression. Gastroenterology.

[7] Nuzzo G, Clemente G, Cadeddu F, Ardito F, Ricci R, Vecchio FM (2005). Papillary carcinoma of the gallbladder and anomalous pancreatico-biliary junction. Report of three cases and review of the literature. Hepatogastroenterology.

[8] Hundal R, Shaffer EA (2014). Gallbladder cancer: epidemiology and outcome. Clin Epidemil.

[9] Hsing AW, Bai Y, Andreotti G, Rashid A, Deng J, Chen J (2007). Family history of gallstones and the risk of biliary tract cancer and gallstones: a population-based study in Shanghai, China. Int J Cancer.

[10] Konstantinidis IT, Deshpande V, Genevay M, Berger D, Castillo CF-D, Tanabe KK (2009). Trends in presentation and survival for gallbladder cancer during a period of more than 4 decades: a single-institution experience. Arch Surg.

[11] Rawal N, Awasthi S, Dash NR, Kumar S, Das P, Ranjan A (2023). Prognostic relevance of PDL1 and CA19–9 expression in gallbladder cancer vs. inflammatory lesions. Curr Oncol.

[12] Tan Y, Ma SY, Wang FQ, Meng HP, Cuizhu M, Liu A (2011). Proteomic-based analysis for identification of potential serum biomarkers in gallbladder cancer. Oncol Rep.

[13] Orsburn BC (2021). Proteome discoverer—a community enhanced data processing suite for protein informatics. Proteomes.

[14] Szklarczyk D, Gable AL, Lyon D, Junge A, Wyder S, Huerta-Cepas J (2019). STRING v11: protein–protein association networks with increased coverage, supporting functional discovery in genome-wide experimental datasets. Nucl Acids Res.

[15] Chin CH, Chen SH, Wu HH, Ho CW, Ko MT, Lin CY (2014). cytoHubba: identifying hub objects and sub-networks from complex interactome. BMC Syst Biol.

[16] Huang DW, Sherman BT, Lempicki RA (2009). Systematic and integrative analysis of large gene lists using DAVID bioinformatics resources. Nat Protocols.

[17] Fluss R, Faraggi D, Reiser B (2015). Estimation of the Youden Index and its associated cutoff point. Biom J.

[18] Huang HL, Yao HS, Wang Y, Wang WJ, Hu ZQ, Jin KZ (2014). Proteomic identification of tumor biomarkers associated with primary gallbladder cancer. World J Gastroenterol.

[19] Sahasrabuddhe NA, Barbhuiya MA, Bhunia S, Subbannayya T, Gowda H, Advani J (2014). Identification of prosaposin and transgelin as potential biomarkers for gallbladder cancer using quantitative proteomics. Biochem Biophys Res Commun.

[20] Waghmare RS, Kamat RN (2014). Incidental gall bladder carcinoma in patients undergoing cholecystectomy: a report of 7 cases. J Assoc Phys India.

[21] Aishima S, Fujita N, Mano Y, Oda Y, Taketomi A, Shirabe K (2011). Different roles of S100P overexpression in intrahepatic cholangiocarcinoma: carcinogenesis of perihilar type and aggressive behavior of peripheral type. Am J Surg Pathol.

[22] Mathai AM, Alexander J, Huang HY, Li CF, Jeng YM, Fung KM (2021). S100P as a marker for poor survival and advanced stage in gallbladder carcinoma. Ann Diagn Pathol.

[23] Wu Z, Yu X, Zhang S, He Y, Guo W (2022). The role of PI3K/AKT signaling pathway in gallbladder carcinoma. Am J Transl Res.

[24] Parkkila S, Pan PW, Ward A, Gibadulinova A, Oveckova I, Pastorekova S (2008). The calcium-binding protein S100P in normal and malignant human tissues. BMC Clin Pathol.

[25] Zhang L, Zhang H, Yue D, Wei W, Chen Y, Zhao X (2019). The prognostic value of the preoperative albumin to alkaline phosphatase ratio in patients with non-small cell lung cancer after surgery. Thoracic Cancer.

[26] Ohkuma R, Yada E, Ishikawa S, Wada S, Komura D, Kubota Y (2020). High expression levels of polymeric immunoglobulin receptor are correlated with chemoresistance and poor prognosis in pancreatic cancer. Oncol Rep.

[27] Chen YB, Jiang CT, Zhang GQ, Wang JS, Pang D (2009). Increased expression of hyaluronic acid binding protein 1 is correlated with poor prognosis in patients with breast cancer. J Surg Oncol.

[28] Niu M, Sun S, Zhang G, Zhao Y, Pang D, Chen Y (2015). Elevated expression of HABP1 is correlated with metastasis and poor survival in breast cancer patients. Am J Cancer Res.

[29] Wang J, Song Y, Liu T, Pang D, Shi Q, Zhong Z (2015). Elevated expression of HABP1 is a novel prognostic indicator in triple-negative breast cancers. Tumor Biol.

[30] Gao H, Yao Q, Lan X, Li S, Wu J, Zeng G (2016). Elevated HABP1 protein expression correlates with progression and poor survival in patients with gastric cancer. OncoTargets Ther.

[31] Shen X, Han B, Shen Y, Ren T, Sha G, Xiang Y (2014). Expression of C1QBP gene and its correlation with drug resistance in human resistance choriocarcinoma cell line. Zhonghua fu Chan ke za zhi.

[32] Assinder SJ, Stanton J-AL, Prasad PD (2009). Transgelin: an actin-binding protein and tumour suppressor. Int J Biochem Cell Biol.

[33] Shields JM, Rogers-Graham K, Der CJ (2002). Loss of transgelin in breast and colon tumors and in RIE-1 cells by Ras deregulation of gene expression through Raf-independent pathways. J Biol Chem.

[34] Thompson O, Moghraby JS, Ayscough KR, Winder SJ (2012). Depletion of the actin bundling protein SM22/transgelin increases actin dynamics and enhances the tumourigenic phenotypes of cells. BMC Cell Biol.

[35] Tsui KH, Lin YH, Chang KS, Hou CP, Chen PJ, Feng TH (2019). Transgelin, a p53 and PTEN-upregulated gene, inhibits the cell proliferation and invasion of human bladder carcinoma cells in vitro and in vivo. Int J Mol Sci.

[36] Yamane T, Asanoma K, Kobayashi H, Kato K, Liu G, Yagi H (2015). Identification of the critical site of calponin 1 for suppression of ovarian cancer properties. Anticancer Res.

[37] Lin Z-Y, Chuang W-L (2012). Genes responsible for the characteristics of primary cultured invasive phenotype hepatocellular carcinoma cells. Biomed Pharmacother.

[38] Mamoor S.

[39] Hammad A, Elshaer M, Tang X (2021). Identification of potential biomarkers with colorectal cancer based on bioinformatics analysis and machine learning. Math Biosci Eng.

[40] Chen M, Li S, Topatana W, Cai X, Lv X, Cao J (2021). Development and validation of a nomogram for predicting survival in gallbladder cancer patients with recurrence after surgery. Front Oncol.

[41] Higuchi R, Yazawa T, Uemura S, Yamamoto M, Ota T, Araida T (2020). Examination of prognostic factors affecting long-term survival of patients with stage 3/4 gallbladder cancer without distant metastasis. Cancers.

[42] Uzun MA, Tilki M, Kayaoğlu SA, Okuyan GÇ, Kılıçoğlu ZG, Gönültaş A (2022). Long-term results and prognostic factors after surgical treatment for gallbladder cancer. Turkish J Surg.

[43] Naveed S, Qari H, Thau CM, Burasakarn P, Mir AW, Panday BB (2021). Lymph Node Ratio is an important prognostic factor in curatively resected gallbladder carcinoma, especially in node-positive patients: an experience from endemic region in a developing country. Euroasian J Hepatogastroenterol.

[44] Shirai Y, Sakata J, Wakai T, Ohashi T, Ajioka Y, Hatakeyama K (2012). Assessment of lymph node status in gallbladder cancer: location, number, or ratio of positive nodes. World J Surg Oncol.

[45] Liu Z, Zhu G, Jiang X, Zhao Y, Zeng H, Jing J (2020). Survival prediction in gallbladder cancer using CT based machine learning. Front Oncol.

[46] Sachan A, Saluja SS, Nekarakanti PK, Mahajan B, Nag HH, Mishra PK (2020). Raised CA19–9 and CEA have prognostic relevance in gallbladder carcinoma. BMC Cancer.

[47] Xu WY, Zhang HH, Xiong JP, Yang XB, Bai Y, Lin JZ (2018). Prognostic significance of the fibrinogen-to-albumin ratio in gallbladder cancer patients. World J Gastroenterol.

[48] Bandırmalı O, Yilmaz Z, Arikan TB (2020). Prognostic factors in gallbladder cancer. Erciyes Med J.

[49] Farhat MH, Shamseddine AI, Tawil AN, Berjawi G, Sidani C, Shamseddeen W (2008). Prognostic factors in patients with advanced cholangiocarcinoma: role of surgery, chemotherapy and body mass index. World J Gastroenterol.

[50] Tran TB, Norton JA, Ethun CG, Pawlik TM, Buettner S, Schmidt C (2017). Gallbladder cancer presenting with jaundice: uniformly fatal or still potentially curable?. J Gastrointestinal Surg.

